# Federated learning with integrated attention multiscale model for brain tumor segmentation

**DOI:** 10.1038/s41598-025-96416-6

**Published:** 2025-04-07

**Authors:** Sherly Alphonse, Fidal Mathew, K. Dhanush, V. Dinesh

**Affiliations:** School of Computer Science and Engineering, Vellore Institute of Technology, Chennai, India

**Keywords:** Federated learning, Mixed-FedUNet, UNet, Segmentation, Images, Image processing, Machine learning

## Abstract

Brain tumors are an extremely deadly condition and the growth of abnormal cells that have formed inside the brain causes the illness. According to studies, Magnetic Resonance Imaging (MRI) is a fundamental imaging method that is frequently used in medical diagnostics to identify, treat, and routinely check for brain cancers. These images include extremely private and delicate details regarding the brain health of the individuals and it must be treated with much care to ensure anonymity of patients. However, traditional brain tumor segmentation techniques usually rely on centralized data storage and analysis, which might result in privacy issues and violations. Federated learning offers a solution by enabling the cooperative development of brain tumor segmentation models without necessitating the transfer of raw patient data to a centralized location. All the data are held securely within their institution. A Reinforcement Learning-based Federated Averaging (RL-FedAvg) model is proposed that fuses the Federated Averaging (FedAvg) model with Reinforcement Learning (RL). To optimize the global model for image segmentation jobs as well as to govern the consumption of client resources, the model dynamically updates client hyperparameters upon real-time performance feedback. A Double Attention-based Multiscale Dense-U-Net model, known as mixed-fed-UNet, is proposed in the work that uses the RL-FedAvg algorithm. The proposed technique achieves 98.24% accuracy and 93.28% dice coefficient on BraTs 2020 dataset. While comparing the developed model with the other existing methods, the proposed methodology shows better performance.

## Introduction

Segmentation of brain tumors constitutes a crucial area in the broad category of medical image analysis as it allows for proper identification and follow-up of patients affected by brain tumors. It constitutes the activity of identification and outlining of abnormal tissue areas in images of the brain, which helps clinicians determine tumor size, shape, and location. Deep Learning (DL) is now recognized as a powerful method of brain tumor segmentation based on its ability to learn discriminative features, even without having to scan through large datasets^1–3^. This implies that the efficacy of deep learning models for the segmentation of brain tumor entities is highly dependent upon the quality of the training of annotated data. Compared with other domains of images, where large-scale datasets are readily available, the acquisition of annotated medical imaging datasets is complicated particularly in the case with brain tumors. The challenge lies in the fact that it requires manual identification and differentiation of the regions of the tumors by qualified radiologists or physicians. This process is often very challenging and time-consuming. Complexities and heterogeneities related to brain tumor images regarding various sizes, shapes, locations, and imaging techniques also compound the difficulty of acquiring and annotating datasets.

**Fig. 1:**
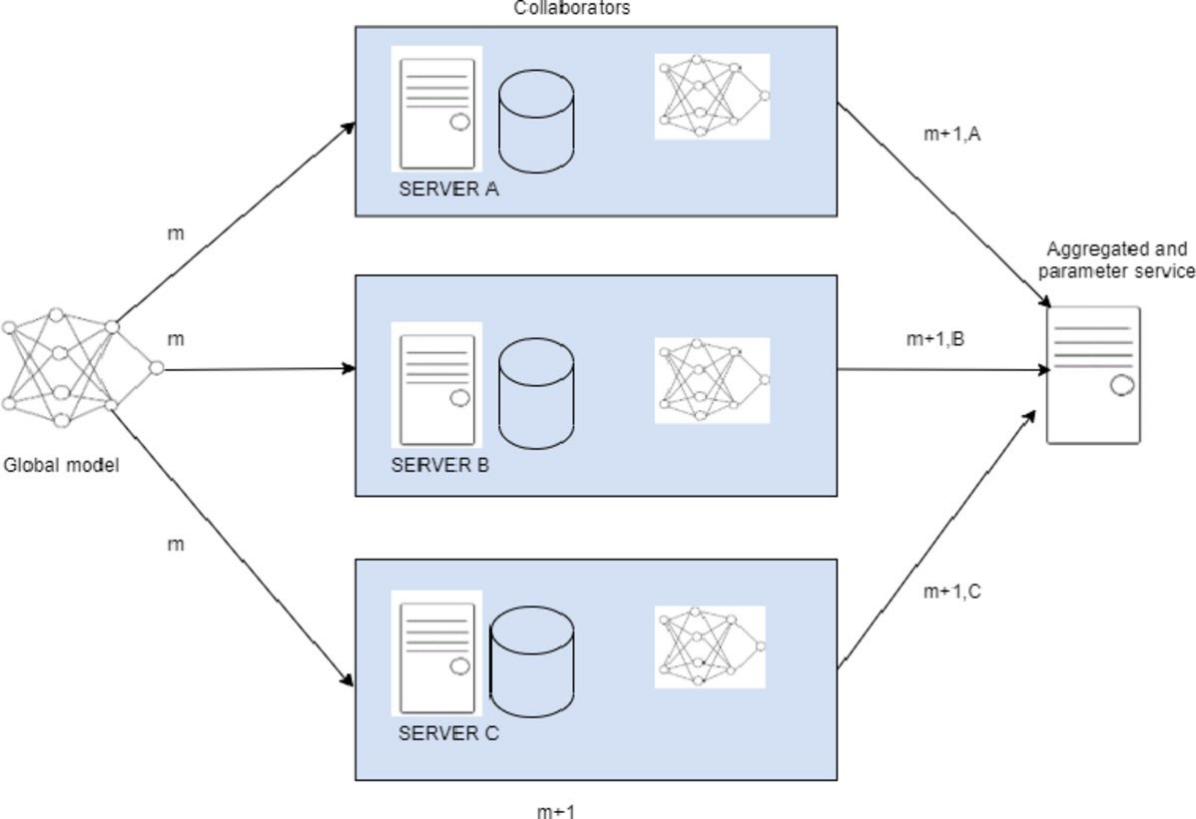
System architecture of Federated Learning.

In Federated Learning (FL), a client uses local data for training its model and it sends only updates of the model to the central server as in Fig. 1. The aim of clients is to train on local data and use it for developing their respective models. The server aggregates and computes the entire weights of all the models to create a global model and sends it to the clients for further training after updating the shared parameters. In that case, every client’s training data is ensured to remain on his or her site and kept confidential throughout the learning process. The only information sent to the central server is the updates of the model, which protects the privacy of the patients and enables inter-institutional cooperation. Within the proposed approach, the segmentation of the brain tumor images from the BraTS2020 dataset is carried out using the UNet architecture. The application of the Federated Averaging (FedAvg) combined with Reinforcement Learning (RL) for the optimization of clients in image segmentation can be visualized by considering the server as some kind of RL agent. It optimizes specific client-specific hyperparameters, such as the learning rate, batch size, or number of local epochs. The objective is the dynamic adjustment of such parameters for better performance of the model in conjunction with RL-FedAvg. This research article explores the method in which FL provides the privacy alongside the accuracy to perform the task of segmentation. This work is, therefore intended to achieve the following goals.

• To design a new model on FL that increases the precision of the brain tumor segmentation while being privacy preserving about the data.
• To implement a framework for brain tumor segmentation based on an appropriate U-Net model.
• Improvement in feature extraction, performance, and localization using the proposed hybrid Double Attention-based Multiscale Dense-U-Net model along with FL known as mixed-fed-UNet in this work.
• Implementation of RL-FedAvg in the model to adapt client-specific hyperparameters dynamically.
•The model is being evaluated for performance using assessment measures such as accuracy, dice coefficient, MeanIOU, precision, sensitivity, and specificity.

This paper aims to provide an in-depth analysis of the advantages, limitations, and practical implications of FL for brain-tumor segmentation. It is focused on three main interests: accuracy, privacy, and scalability. The accuracy of image segmentation can be improved by extending the FL-based U-Net model for multi-scale learning. The proposed Double Attention-based Multiscale Dense-U-Net within the FL framework for image segmentation would address two main areas: efficiency in segmentation tasks and inherent privacy concerns with distributed data models.

The paper is structured into the following ways. In Sect. "Literature review", an extensive review of literature of the study subject is given. Section "Methodology" describes the preprocessing technique, the Double Attention-based Multiscale Dense-U-Net architecture and the RL-FedAvg algorithm. A set of results in Sect. "Experiments and results" demonstrates the performance improvement using the proposed model. Ultimately, the Sect. "Conclusion" provides a summary overview of the findings of research that identifies very critical areas in need of further scholarly review.

## Literature review

Significant progress has been made in the recent years for brain tumor segmentation and medical image privacy. Studies have employed a variety of techniques to enhance precision in their work and to improve privacy of their models for various use cases. This section looks at some of the appropriate field studies and discusses the advantages and drawbacks of different approaches. The authors in^1^ implemented an automated glioblastoma segmentation method using specialized Deep Neural Networks (DNNs), integrating local-global feature Convolutional Neural Network (CNN) architectures and a two-phase training strategy to tackle label imbalance in tumor data. The design is nearly 30 times quicker than the previously published state-of-the-art, according to findings reported on the 2013 BraTS test dataset.

In a FL setting, Li et al.^2^ examined the viability of incorporating methods of differential privacy to safeguard the confidentiality of patient information. Their study utilizes the BraTS2018 dataset, a widely recognized benchmark dataset in the realm of medical image processing. They forced differential privacy approaches within the federated learning framework. Li et al. aimed to ensure that sensitive patient information stayed safe and confidential all through the model training action. Noori et al.^3^ created a 2D UNet-based network with minimal parameters, which integrated attention mechanisms and multi-view fusion while employing adaptive channel weighting and 3D contextual information within a 2D model architecture. Experimental data showed that their solution outperformed leading methods from 2017 to 2018.

Bercea et al.^4^ developed FedDis, a novel federated approach that separates Magnetic Resonance Imaging (MRI) image parameters into shape and appearance. It shares only shape details across clients for privacy preservation, ensuring healthy brain scans to identify abnormalities, and achieving superior performance (average dice: 0.38) on multiple sclerosis (MS) Lesions and MS/Glioblastoma databases, surpassing state-of-the-art methods by 42% (auto-encoder) and 11% (federated method). Islam et al.^5^ presented a work that offers a 3D attention-based UNet for brain tumor segmentation and predicted patient survival days by integrating shape, geometrical, and clinical information. It used training regression approaches using Support Vector Machine (SVM), Artificial Neural Network (ANN), Random Forest and Extreme Gradient Boosting (XGBoost) to evaluate performance.

Luu et al.^6^ improved the previous model, nn-UNet, by incorporating a larger network, group normalization, and axial attention in the decoder, demonstrating efficacy through internal 5-fold cross-validation and organizer-conducted evaluations for minor metric improvements over the baseline. Ummadi^7^ presented the HoMeNL (Homogeneous Region Selection with Kernel Density Estimation and Non-Local Filtering) filter, which employed a manual selection of homogeneous regions and KDE (Kernel Density Estimation) for previous statistical parameter estimates, to enhance (Pol)(In)SAR (Polarimetric (Interferometric) Synthetic Aperture Radar) image filtering. The proposed HoMeNL filter improved the tradeoff estimate without patch-like artifacts while maintaining point targets, proving its denoising efficacy.

Parekh et al.^8^ looked at the applications of FL across a range of domains, with a focus on object detection and segmentation in multimodal, multi-organ contexts. It produced encouraging results (overlap similarity: 0.79 for organ localization, 0.65 for lesion segmentation), demonstrating the viability of multi-domain models without data sharing. Wicaksana et al.^9^ introduced FedMix, a federated learning framework for medical image segmentation that is independent of labels. FedMix incorporated various label types from client data and dynamically modified aggregate weights. The proposed FedMix algorithm demonstrated superior performance compared to current leading methods in the field by a substantial margin, in two complex medical image segmentation tasks like delineating breast tumors and identifying skin lesions.

In a study, Joseph et al.^10^ suggested segmenting brain MRI images using the K-means clustering algorithm in conjunction with morphological filtering to minimize wrongly clustered regions that are unavoidable following segmentation of the brain MRI image for tumor location detection. Li et al.^11^ evaluated the uses of FL in many sectors, to guide future industrial engineering applications and recommended six research fronts to optimize FL comprehension and development.

Tedeschini et al.^12^ presented a real-time distributed networking framework for healthcare-based FL that used both parameter server and decentralized consensus-driven paradigms. The validity of the framework is demonstrated through its application in the segmentation of brain tumors, utilizing a customized U-Net model and clinical datasets obtained from machines located in various geographical locations. It used FL across geographically distributed workstations via the internet.

The authors^13^ in this study introduced a technique for segmenting brain tumors that controls 3D contextual data. The work introduced a unique loss function that mixed Dice and cross-entropy losses to maximize segmentation performance. The system was evaluated in detail using the BraTS 2018 dataset, a well-known standard in medical imaging research. The authors used nearby slices to increase the accuracy of single-mode MRI pictures. The system segmented brain tumors well, as seen by its remarkable average Dice score of 0.88. This research significantly advanced the field of medical image processing by demonstrating how new loss functions and 3D contextual information can enhance segmentation accuracy.

Xu et al.^14^ presented an automatic low-grade glioma MRI scan segmentation utilizing UNet + + with data normalization with a dropout integrated after each convolution layer to reduce overfitting during model training. The proposed model obtained an average Dice coefficient of 89.1% on tumor images in the testing data, indicating superior automatic segmentation for low-grade glioma images and providing valuable diagnostic and surgical guidance to medical practitioners.

Mostafa et al.^15^ proposed a deep learning-based approach. In order to include incomplete client input in diverse contexts, AdaFedProx^16^ employed a reinforcement learning-driven approach, adding a customized proximal term into the objective function. AdaFedProx is able to make regularized judgments throughout the federated training process by dynamically identifying the proximal term based on individual client conditions, including crucial features like data distribution, system specification, and performance feedback. In order to include incomplete client input in diverse contexts, AdaFedProx employed a reinforcement learning-driven approach, adding a customized proximal term into the objective function. AdaFedProx is able to make regularized judgments throughout the federated training process by dynamically identifying the proximal term based on individual client conditions, including crucial features like data distribution, system specification, and performance feedback. Existing approaches used a pre-trained CNN on the BraTS^17^ dataset with categorical cross-entropy loss and the Adam optimizer to successfully segment brain tumors. This approach had a validation accuracy of 98%. CNN also performed exceptionally well in cell tracking tasks for transmitted light microscopy pictures, segmenting 512 × 512 images in less than a second on today’s GPU^18,19^.

Lee et al.^20^ introduced a novel method that combines Federated Machine Learning (FML) with traditional Machine Learning (ML) methods to classify fetal status based on cardiac data. The FML model achieved an impressive prediction rate of 99.06% with an incorrect prediction rate of 0.94%, compared to its proven performance. In this study, K-nearest neighbor (KNN) was used, achieving an accuracy of 82.93% with an incorrect prediction rate of 17.07%. This establishes the power FML has in increasing the accuracy and reliability of fetal health assessment in pregnancy.

Moshawrab et al.^21^ investigated FL to address privacy concerns in healthcare and finance, including sensitive data. FL protected privacy while enhancing performance by exchanging parameters rather than raw data during model training. The study examined FL’s technical elements, how it differs from standard ML, and existing aggregation algorithms. It also looked at how FL can be used to diagnose diseases like cardiovascular disease, diabetes, and cancer. The paper also discussed the obstacles to FL’s advancement and provided ways for overcoming these constraints to promote its adoption in diverse sectors.

Tan et al.^22^ presented an integrated learning framework for breast cancer screening that addressed the privacy issues associated with centralized learning. It used transfer learning, SMOTE (Synthetic Minority Over-sampling Technique) data processing, and FeAvg-CNN (Feature Averaging Convolutional Neural Network) + MobileNet in the FL framework. The results showed high classification performance, emphasizing the potential of FL to improve AI healthcare delivery. Subashchandrabose et al.^23^ provided a FL algorithm for multi-level lung cancer classification, addressing the limitation of single modeling approaches due to centralized data limitations. Improved analysis of the Kaggle cancer dataset gave encouraging results, with an accuracy of 89.63% in lung cancer classification.

Xu et al.^24^ introduced FedSM, a novel training framework for cross-silo FL in medical image classification tasks. FedSM addressed the issue of customer visits and generalization gaps by proposing individual FL values ​​and using the SoftPull method. Rigorous theoretical analysis ensured convergence for optimizing non-convex smooth objective functions. The model has been validated through experiments in real-life scenarios, demonstrating its capability to improve medical image segmentation with the use of deep FL techniques. Federated Cross Learning (FedCross) technique was used to overcome the problem of non-iid medical image data in FL. It outperformed traditional FL methods by training the global model sequentially across many clients without model aggregation. Additionally, FedCross’s effectiveness and clinical relevance in medical image analysis are demonstrated by FedCross’s combination with ensemble learning in Federated Cross Ensemble Learning (FedCrossEns), which enhances segmentation performance.

Yang et al.^25^ proposed private FL. Dasha et al.^26^ presented the FL for covid images. Guan et al.^27^ provided a detailed evaluation of latest developments in FL applied to medical image analysis, categorizing known FL algorithms and explaining their principles using a “question-answer” paradigm. It demonstrated the usefulness of various FL techniques using empirical comparisons on benchmark datasets, identified present problems, and outlined future research objectives. It seemed to encourage additional research and innovation in health sector.

Adnan et al.^28^ examined the influence of FL techniques (concurrent, semi-concurrent, incremental, and cyclic-incremental) on global model performance across a variety of federated setups, providing insights for optimizing FL implementations. Understanding these dynamics, the development of specialized FL systems can improve collaborative learning across distributed edge nodes. Sohan et al.^29^ investigated FL’s use in medical picture analysis, focusing on its decentralized data processing for increased privacy in AI. This work provided vital insights for enhancing healthcare AI methodology by comparing it to typical ML models and discussing the issues it faces. By combining various study findings, the paper provides a comprehensive resource for understanding FL’s significance in medical imaging and directing future research efforts.

Sachin et al.^30^ presented FedCure, a tailored FL framework built specifically for intelligent Internet of Medical Things (IoMT) applications in a cloud-edge architecture. FedCure effectively solves IoMT’s complicated variations by using tailored FL approaches to alleviate heterogeneity problems. FedCure enhances the rate at which data is processed and diminishes the delay in Internet of Medical Things (IoMT) applications, as evidenced by case studies in a variety of healthcare sectors. Overall, FedCure performs admirably in tackling IoMT difficulties, providing accuracy with low communication overhead, and demonstrating its potential to advance intelligent healthcare systems. The Table 1 describes the limitations from previous studies.

**Table 1:** Limitations from previous studies.

Reference	Model	Dataset	FML	Results	Limitation
^1^	Specialized DNNs with local-global CNN	BRATS 2013	No	Improved over state-of-the-art techniques and is 30x faster	Specific to BRATS 2013 dataset, focusses on glioblastoma
^2^	Differential privacy in federated learning	BRATS 2018	Yes	Privacy protection and secured model training	Limited to BRATS 2018 dataset
^4^	FedDis	MS Lesions, MS/Glioblastoma	Yes	Superior performance and privacy-preserving	Specific to federated learning, may lack generalization
^5^	3D attention-based UNet	BRATS 2019	No	Outperformed previous models	Requires higher computational resources and 3D modeling
^10^	K-means clustering and morphological filtering	Brain MRI	No	Improved segmentation for tumor detection	Limited to K-means algorithm
^13^	3D contextual information with unique loss function	BraTS 2018	No	Dice score of 0.88	Focuses on specific data and lacks privacy
^15^	Pre-trained CNN	BraTS 2017	No	Validation accuracy of 98%	Limited dataset and privacy

This manuscript aims to rectify the drawbacks present in previous methodologies used for the segmentation of brain tumors. This paper focused on privacy enhancement and collaboration to allow data to remain at the different institutions while the central server only receives updates on model changes. Most of the existing works does not involve maintaining privacy and only focuses on accuracy. A novel FL architecture known as mixed-fed-UNet is presented in this paper. It allows to preserve privacy. In contrast to previous studies that relied on older datasets, this approach acknowledges the importance of utilizing current data to enhance the effectiveness and generalizability of segmentation models. This work prioritizes using a more recent dataset, BraTS 2020^17^, to ensure the relevance and applicability of our findings. This enhanced dataset, advances the investigation of FL algorithms for brain tumor segmentation tasks. The use of BraTS 2020^17^ allows for a more comprehensive knowledge of the issues inherent in brain tumor segmentation and the creation of increasingly reliable and precise models. Furthermore, this proposed approach encourages further investigation of official approaches for medical imaging. FL addresses the privacy challenges associated with centralized data processing by decentralizing the ideal training process and prioritizing privacy protections. Through studies and testing it is hoped to discover the ability of FL to meet the unique needs of brains to address tumor segmentation while maintaining patient confidentiality and data security.

## Methodology

**Fig. 2:**
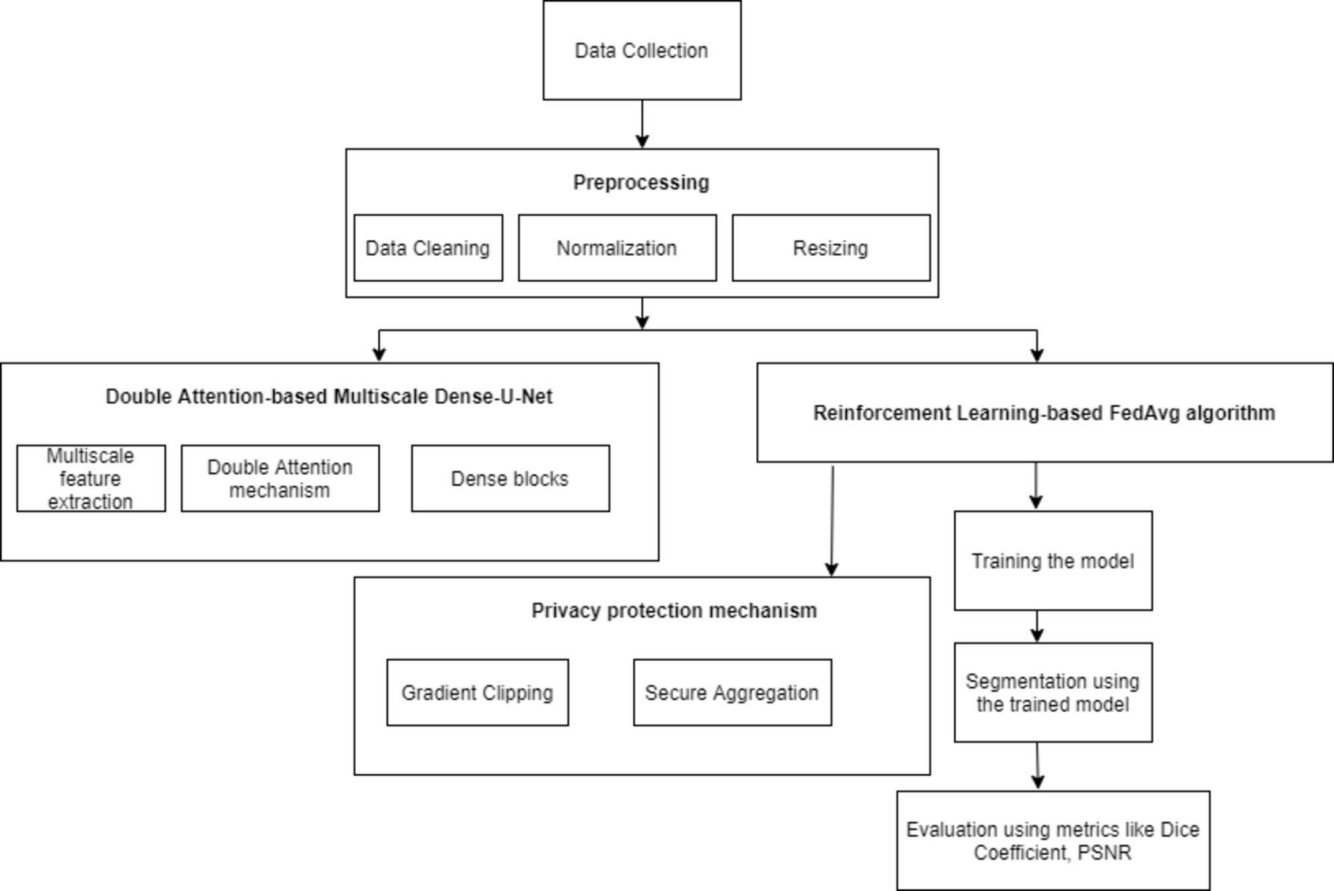
Roadmap of the proposed technique.

This section offers a comprehensive summary of the proposed research and also outlines the procedures for data preparation, preprocessing, and the architecture of the UNet model that uses FL. The Fig. 2 illustrates the proposed methodology where the data is preprocessed and given to the U-Net model using FL for segmentation.

The roadmap given in Fig. 2 describes the detailed, step-by-step procedure for developing, training, and deploying an advanced brain image segmentation model with a high level of privacy protection and optimization through reinforcement-based FL. The preprocessing of the images is done and given to the Double Attention-based Multiscale Dense-U-Net along with RL-FedAvg algorithm. Such an approach may impact clinical workflows in a crucial way by being accurate and available in real-time for brain tumor diagnosis and treatment planning. Differential Privacy is used to ensure that local data cannot be reverse engineered from updated aggregated models. Gradient Clipping limits the L2 norm of the gradients such that very large updates wouldn’t occur, which might leak local data information. Update aggregation is done on the server without exposing individual model updates from devices that participated. Evaluation Metrics like Dice Coefficient is applied to measure accuracy of segmentation. Also, a higher PSNR indicates less inversed.

### Preprocessing

In this work, the Keras sequence DataGenerator class is overridden to match the requirements. The `DataGenerator` class orchestrates the loading, resizing, and normalization of brain MRI images. Each MRI image, consisting of multiple modalities like Fluid-Attenuated Inversion Recovery (FLAIR) and T1-Contrast-Enhanced (T1-CE), is loaded from the specified file paths using Neuroimaging Informatics Technology Initiative (NIfTI) file format utilities. The images are then resized to a uniform dimension using OpenCV, ensuring consistency across the dataset. Normalization is applied to standardize the intensity values of the images, a crucial step for enhancing model convergence during training. By encapsulating these preprocessing steps within the `DataGenerator`, the class streamlines data preparation, ensuring that input data is appropriately formatted and optimized for subsequent training, validation, and testing stages. This streamlined preprocessing workflow enhances the model’s ability to learn meaningful features from the data, ultimately leading to improved segmentation performance.

### Federated learning

In traditional machine learning or middle learning, all participating organizations (collaborators) share their training data with a centralized server. In contrast, in FL, participants train a locally shared model and communicate only updates to a central server, rather than sharing their data. The server collects and combines any changes to create a global model, after which it sends updated shared parameters to each client for further training. The FL technique enables a learning approach that trains algorithms in unity without sharing the underlying datasets, aiming to solve data governance and privacy issues. This eliminates the need for data storage and delivery in one location. Multiple collaborators in different areas can generate identical, reliable ML models. As a result, important issues such as data protection, privacy, copyright, and the use of heterogeneous data are discussed.

**Fig. 3:**
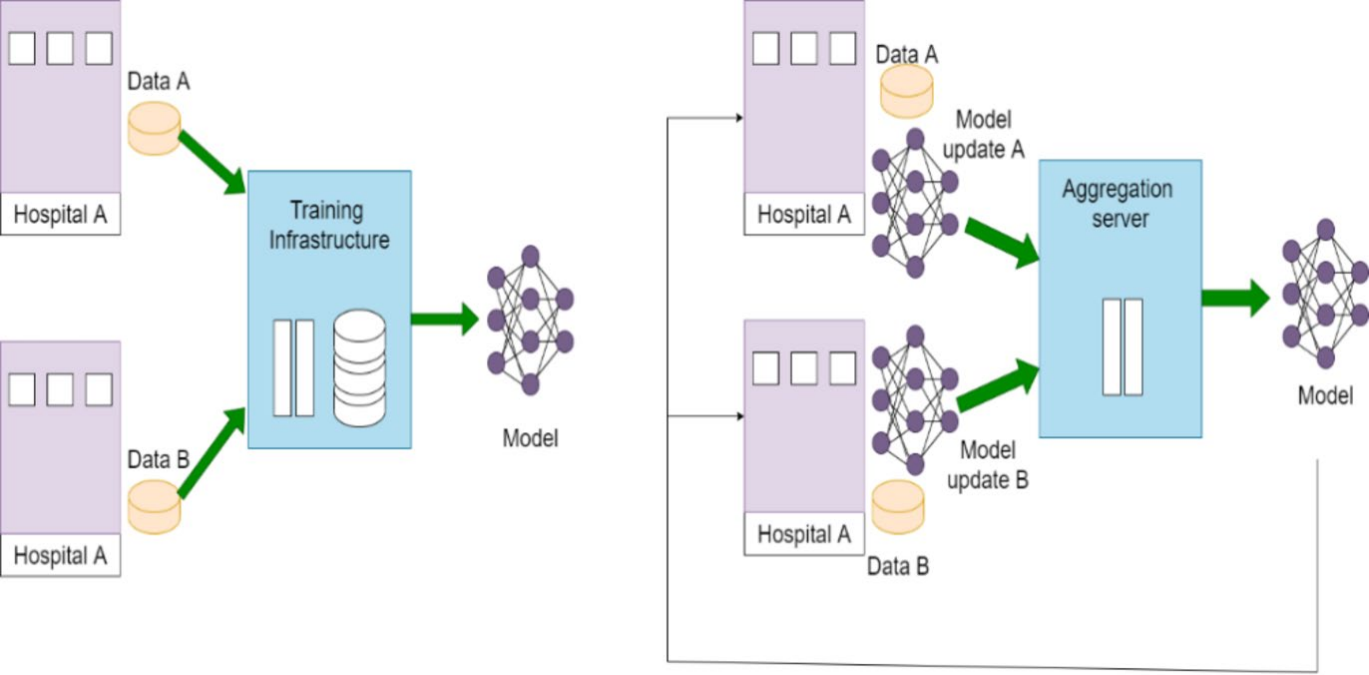
Centralized Learning vs. Federated Learning.

Figure 3 illustrates the Centralized Learning and FL. In Centralized Learning the data is stored in one location, making it easier to preprocess and ensure consistency. In FL the devices often have non-identical, unevenly distributed, and even biased data (referred to as non-iid data). Handling such heterogeneity can be challenging and may affect model performance. The ability of FL to protect patient privacy has attracted considerable attention in the healthcare industry. Some trust is still needed in the central server responsible for client training^31,32^. A few extra elements are added to the conventional centralized training process by federated learning like:

1. collaborator

A FL algorithm has multiple users collaborating simultaneously to train the global model. Each entity is known as a client and is just a part of the consortium that has access to any of the client’s local data.

2) parameter server

In a FL system, the parameter/central server controls the training process. It distributes copies of the global model to its colleagues. Parameter servers and aggregators are typically associated with a single computing node.

3) aggregator

The aggregator collects customized local images from contributors and combines them into a new global model. Federated averaging, also known as weighted averaging, is often used to combine locally generated models.

4) round

A federation round is an interval of training stages that involves aggregating. Collaborators can train the model locally for several epochs, including incomplete epochs, within a single training round. The typical procedure for training a FL system consists of the following stages:

(i) The collaborator receives global model updates from the server, trains on personal data, and then sends local model changes to the global server.
(ii) After receiving the updated weights of the local model, the central server securely aggregates the data without gaining knowledge about any collaborators, producing a global model.
(iii) The collaborators update it locally after receiving the revised shared weights from the central server for further training.
(iv) Go back to i) for another federated round.

**Fig. 4:**
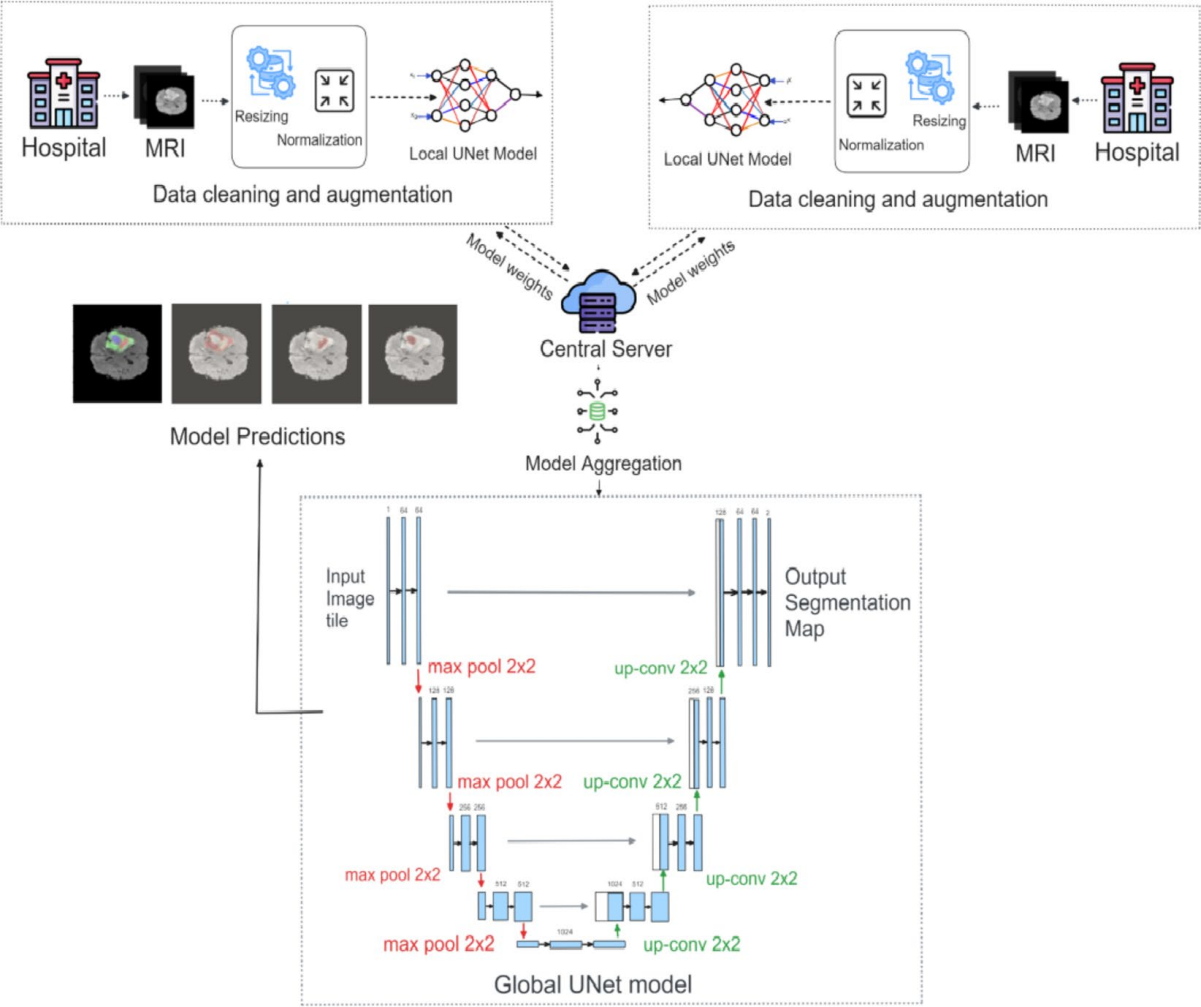
Overall architecture with global U-Net model.

Figure 4 demonstrates the architecture of the proposed technique with conventional U-Net. In FL, it is considered that the dataset is spread across clients $\:m=1,\:2,\ldots M$. The notation is as follows.

There is a set of indices (Pm) of data points that signify each division for each client. Here, $\:n$ is the total number of data points gotten by all clients, and $\:nm$ designates the number of data points that the client currently owns, and nm = |Pm|. In this federated scenario, in mini-batch stochastic gradient descent, the calculation work for a single complete update is identified by three factors. The formula used for minimizing a loss function is indicated below:

1 $$\:f\left(\theta\:\right),\:where{\:{f\left(\theta\:\right)}^{ghi}}^{\:}=\:{\sum\:}_{b}^{a}{f}_{i}\left(\theta\:\right)$$

Then, each client receives a step of gradient descent and informs its settings appropriately, dignified as:

2 $$\:f\left(\theta\:\right)\:=\:{\sum\:}_{a=1}^{A}\frac{{n}_{a}}{n}{F}_{a}\left(\theta\:\right)$$

3 $$\:\text{W}\text{h}\text{e}\text{r}\text{e},\:{F}_{a}\left(\theta\:\right)\:=\:\frac{1}{{n}_{a}}{\sum\:}_{i}^{\:}{f}_{i}\left(\theta\:\right)\:\:$$

The average loss for a stated client is computed as:

4 $$\:\frac{1}{{n}_{a}}{\sum\:}_{\:}^{\:}i\epsilon {p}_{a}{f}_{i}(\theta)$$

Now, let $\:{\theta\:}_{t}^{i}$ represent the model parameters of client $\:i$ at iteration $\:t$, and $\:{N}_{i}$ be the number of samples available at client $\:i$. Then, the local update performed by a client $\:i$ can be represented as:

5 $$\:{\theta}_{t+1}^{i}={\theta}_{t}^{i}-\:\eta\triangledown{f}_{i}\:({\theta}_{t}^{i})$$

Where η is the learning rate and $\:{f}_{i}$($\:{\theta\:}_{t}^{i}$) is the local loss function at client $\:i$ with model parameters $\:{\theta\:}_{t}^{i}$.

The server uses average to aggregate the model updates, once each client completes their local update:

6 $$\:{\theta\:}_{t+1}^{\:}\:=\:{\sum\:}_{i=1}^{K}\frac{1}{N}\:{\theta\:}_{t+1}^{i}$$

Where $\:N$ is the total number of clients. In the proposed mixed-Fed-U-Net,

- Local models are trained independently on their respective datasets.
- The global model is constructed using FedAvg, which takes a weighted average of the weight of local models.
- The global model is updated and distributed to all participants for the next training round.

### Mathematical model of federated learning with RL optimization (RL-FedAvg)

The Reinforcement Learning (RL)^4,33^ is incorporated in Federated averaging model as follows:

Step 1: The global model is initialized as follows:

7 $$\:w0=random\:initialization$$

Then the initial global model is distributed among all clients.

Step 2: Client-side training is done with RL-optimized hyperparameters. During every round $\:t$, the central server:

1. Detects the present state $\:{s}_{t}\:$based on the performance of the client and global model metrics.
2. Chooses actions $\:{a}_{t}^{i}=\{{\eta\:}_{t}^{i},{B}_{t}^{i},{E}_{t}^{i}\}$ using the RL policy $\:{\uppi\:}\left({s}_{t}\right)$.
3. Client gets the transmitted optimized hyperparameters from the server

A local training is done by the client as follows:

8 $$\:{W}_{t+1}^{i}={w}_{t}-{\eta\:}_{t}^{i}\nabla\:{L}_{i}{(w}_{t},{D}_{i})$$

Step 3: The reward is computed and the policy is updated. After training, the server assesses the client models depending upon a segmentation metric $\:\text{M}$ and calculates the reward for every client $\:{r}_{t}^{i}$​. The server apprises the RL policy $\:{\uppi\:}\left({s}_{t}\right)$ depending on the reward signal by means of a policy optimization algorithm.

Step 4: Global model is aggregated. The server collections the client models using FedAvg:

9 $$\:{w}_{t+1}=\frac{1}{N}\sum\:_{i=1}^{N}\frac{\left|{D}_{i}\right|}{\sum\:_{i=1}^{N}\left|{D}_{i}\right|}{w}_{t+1}^{i}$$

Step 5: Till convergence the process is repeated. The process is repetitive until the global model attains the wanted segmentation accuracy or until a static number of rounds $\:T$ is finished. For image segmentation, the subsequent metrics can be utilized in the reward function $\:{r}_{t}^{i}$​:

- Dice Coefficient: This is used for measuring the overlap among predicted and ground truth masks.

10 $$\:Dice(P,G)=\frac{2\mid\:P\cap\:G}{\mid\:P\mid\:+\mid\:G\mid\:}$$

where $\:P$ is the projected segmentation mask and $\:G$ is the computed ground truth mask.

- IoU (Intersection over Union): This is a common metric utilized for segmentation tasks.

11 $$\:IoU(P,G)=\frac{\mid\:P\cap\:G\mid\:}{\mid\:P\cup\:G\mid\:}$$

By merging FedAvg with RL-based client optimization, this model guarantees dynamic tuning of client hyperparameters based on real-time feedback, thereby optimizing the global model for image segmentation tasks while proficiently handling client resources. The RL-FedAvg algorithm is given as follows:

**Algorithm 1:**
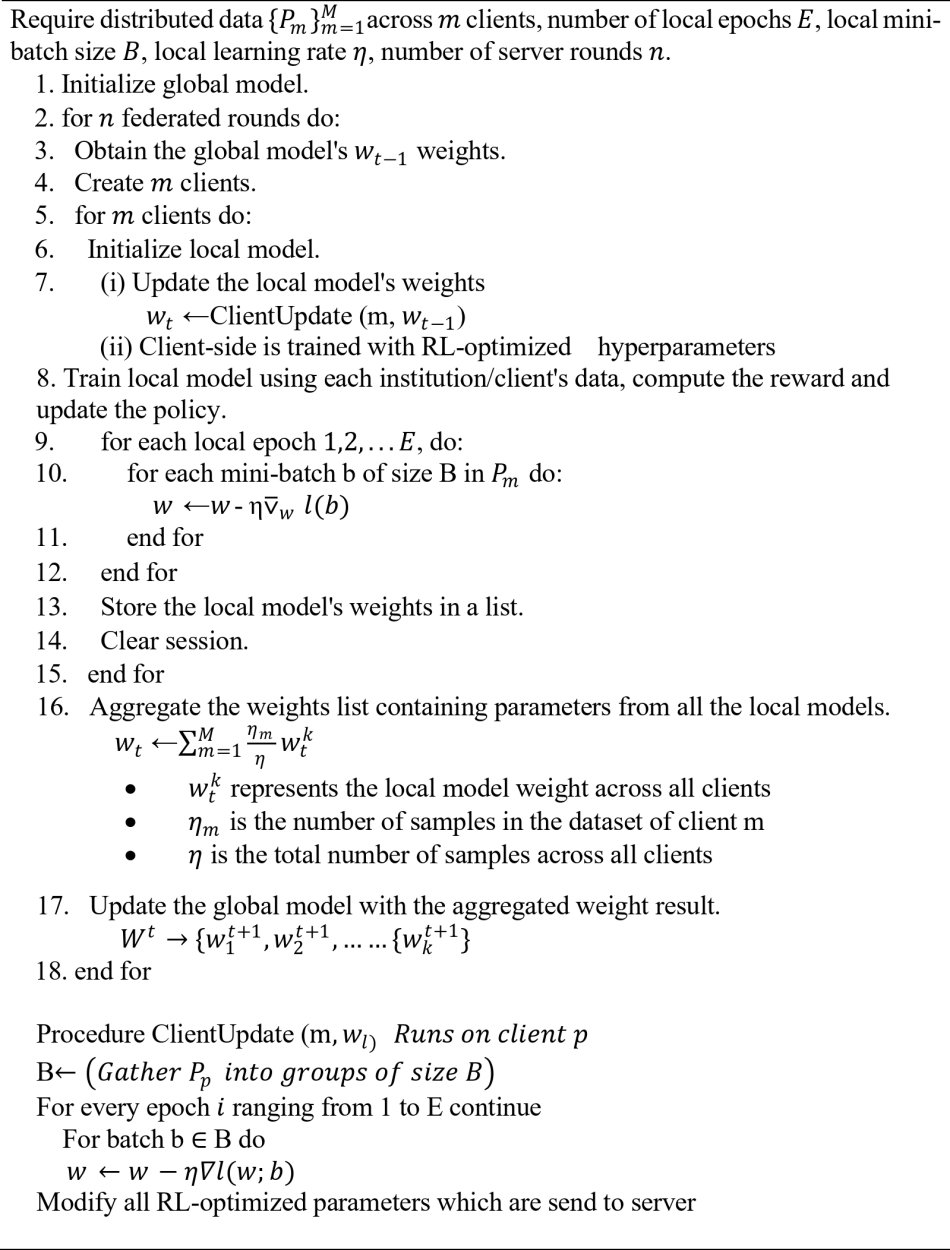
RL-FedAvg in the proposed work.

### Architecture of the proposed double attention-based multiscale Dense-U-Net

The Double Attention-based Multiscale Dense-U-Net is an enhanced model of U-Net. When this proposed model is used instead of the global U-Net, it enhances the performance.

#### Conventional U-Net

Convolutional neural networks such as the U-net architecture are frequently used for image segmentation applications. Figure 4 represents the U-net architecture. It has been especially beneficial in the area of image analysis for medical purposes. The encoder-decoder structure of the model uses skip combinations to preserve spatial information while obtaining both local and global data. High-level features are extracted by the encoder section by successively down sampling the input image using convolutional and max-pooling layers. Two convolutional (Conv2D) layers with 32 filters and Rectified Linear Unit (ReLU) activation precede MaxPooling2D layers ($\:2\times2$ pool size) in the encoder block, which are used for downsampling. Convolutional blocks 64, 128, 256, and 512 are convolutional blocks made up of two Conv2D layers activated by ReLU. The function of activation for ReLU can be expressed mathematically as:

12 $$\:\:f\left(x\right)\:=\:max(0,\:x)$$

As a result, for each given input $\:x$, the output $\:f\left(x\right)$ of the ReLU function equals $\:x$ if $\:x$ is non-negative and zero if $\:x$ is negative. The U-Net architecture can be represented as in Fig. 4.

1. downsampling layers

The number of channels can be augmented while the spatial dimensions of the feature maps are reduced with the incorporation of downsampling layers. Five downsampling layers, two input and one convolutional layer are used in this work. The forward pass of a downsampling layer utilizing convolutional algorithms and pooling is represented mathematically as follows:

13 $$\:{h}_{k}=\:f({W}_{k\:}\times\:\:{h}_{k-1}+{b}_{k}+({W}_{k-1\:}\times\:\:{h}_{k-1}+{b}_{k-1}\left)\right)$$

The forward pass of the $\:k$-th layer in a neural network design is specified by this equation, particularly with regard to downsampling. It requires convolving the input feature map $\:{h}_{k-1}$ with the weights $\:{W}_{k\:}$ of the k-th convolutional layer, adding biases $\:{b}_{k}$, and applying an activation function $\:f.$ The resulting output feature map $\:{h}_{k}$ represents the output of the $\:k$-th layer after downsampling. A residual link from the previous layer captures fine-grained features and improves gradient flow during training.

2) upsampling layers

The upsampling layers are included in this model after downsampling layers. These layers retrieve the fine-grained data which went missing during the downsampling procedure. The forward pass formula of a Conv2D layer is expressed as:

14 $$\:{h^{\prime\prime}}{_{k}}=\:f({W^{\prime\:}}_{k\:}\times\:\:{h^{\prime\:}}_{k-1}+{b^{\prime\:}}_{k}\:+\:({W^{\prime\:}}_{k-1\:}\times\:\:{h}_{N}+{b^{\prime\:}}_{k-1}\:\left)\:\right)$$

The computation performed in the k^th^ layer of the upsampling block is described by the equation above. The (k-1)-th layer’s result, represented by $\:\:{h^{\prime\:}}_{k-1}$, is multiplied by $\:{W^{\prime\:}}_{k\:}$ weights and added by $\:{b^{\prime\:}}_{k}$ biases. The ReLU activation function, represented by the letter $\:f$, is then used to process the output that results. Obtaining the result of the (k-1)-th layer, then multiplying it by the weights $\:{W^{\prime\:}}_{k-1\:}$, and adding the biases $\:{b^{\prime\:}}_{k-1}$ is the first step in this relationship. The resulting downsampling layer, $\:{h}_{N}$, is then mixed with this outcome and fed into the active upsampling layer.

3) final layer

The final layer of the U-Net model produces the output segmentation map together with probability for every class. In this work, the distribution of probability across the classes is generated by our final layer using the softmax activation function. The following represents the mathematical equation to obtain the final layer’s forward pass:

15 $$\:y\:=softmax({W}_{out\:}\text{*}{g}_{1}+{b}_{out})$$

In the above formula, the expected output $\:y$ reflects the segmentation map, with probabilities assigned to each class. The weights of the last layer $\:{W}_{out\:}$ and bias values $\:{b}_{out}$ are applied to the production of the first layer in the decoder block $\:{g}_{1}.$ The softmax activation function then processes the generated logits to provide the final probabilities for each class in the segmentation map. This softmax activation ensures that the projected probabilities add to one over all classes, resulting in probability distributed throughout the classes.

4) Loss function

In order to train the network and minimize the discrepancy between the anticipated segmentation map and the ground truth labels, the loss function of the U-Net model is essential. The loss function used for this task is known as categorically defined cross-entropy, as it deals with numerous classes. For this loss function, the equation is:

16 $$\:L(x,\:y)\:=\:-{\sum\:}_{i}^{\:}{x}_{i}\:log\left({y}_{i}\right)$$

The projected probability for the i^th^ pixel is denoted by $\:{y}_{i}\:$in this notation, whereas $\:{x}_{i}\:$represents the base truth identifier for that pixel. The symbol ∑ indicates the sum over all elements of $\:x$ and $\:y$, allowing for the evaluation of the model’s performance across all pixels. The U-Net model learns to generate accurate mappings indicating the segmentation which closely match the base truth identifiers by minimizing this loss function during training. In addition to the categorically defined cross-entropy loss, the Adam optimizer is also incorporated with the rate of retention and learning set to 0.001.

#### Double attention multiscale Dense-U-Net

**Fig. 5:**
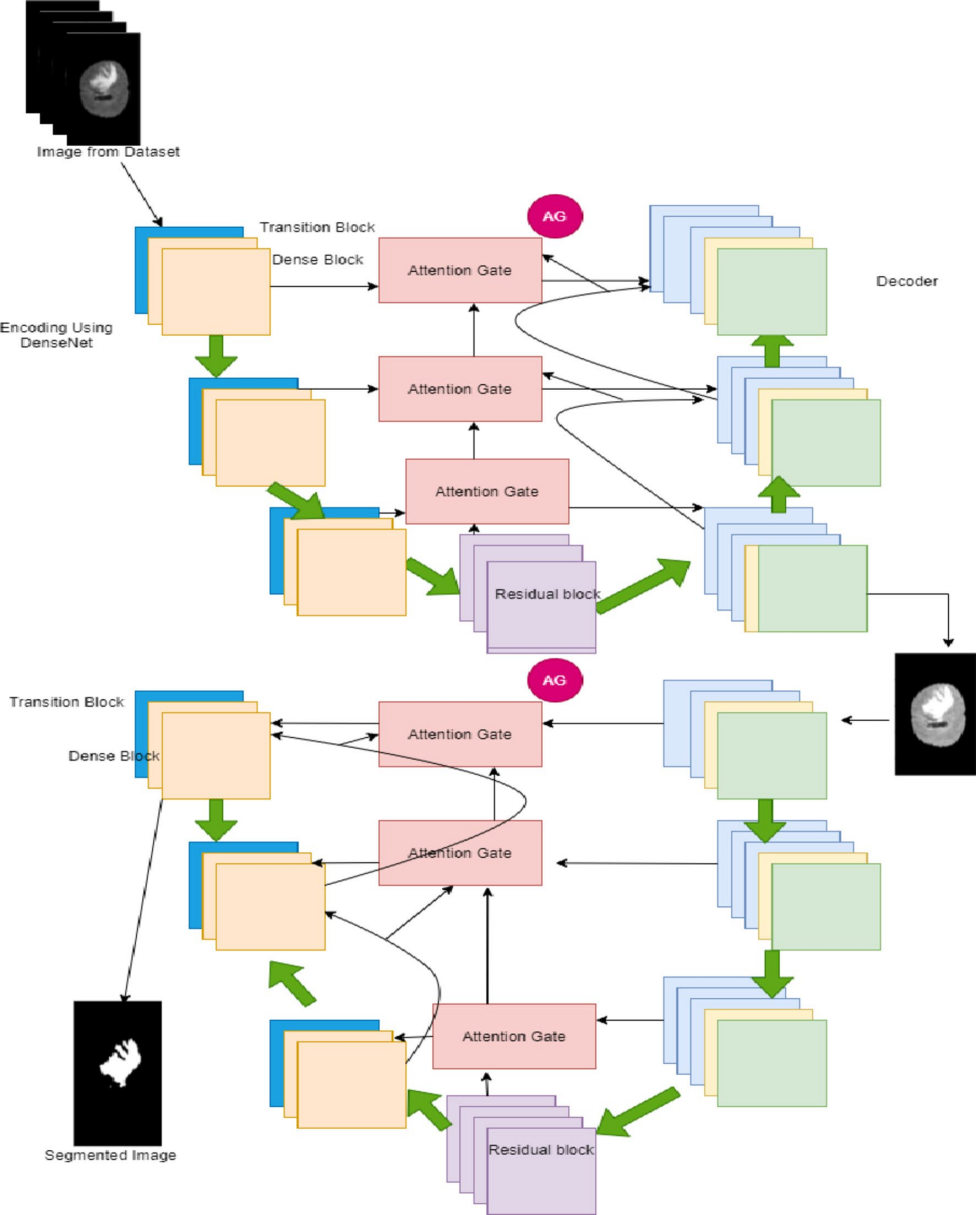
Proposed architecture for UNet.

**Fig. 6:**
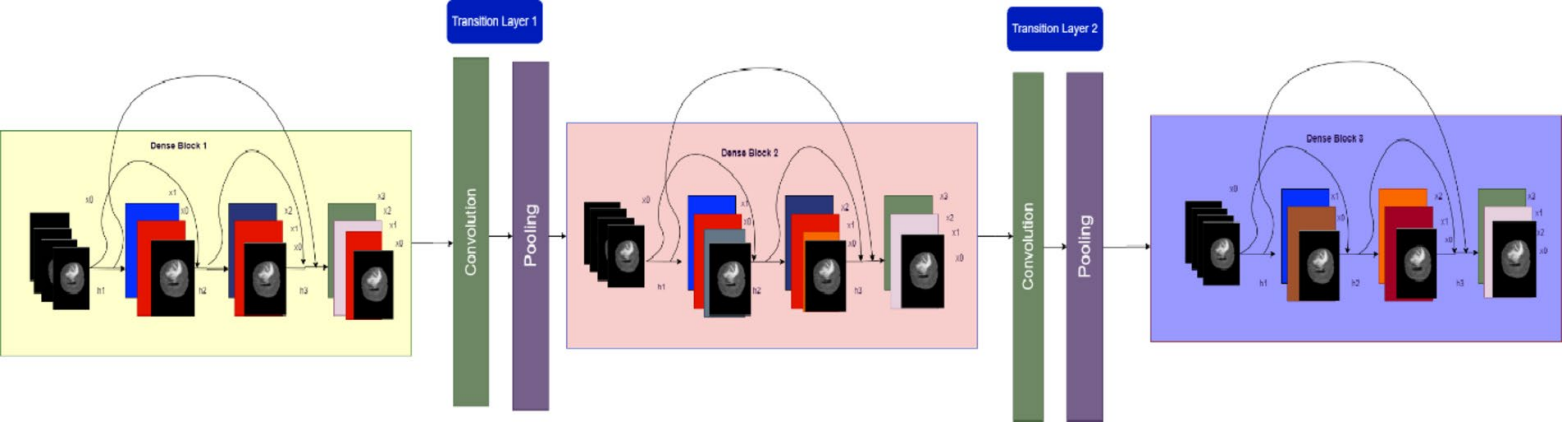
DenseNet.

The proposed segmentation design makes advantage of the U-Net concept^18^, which places an encoder block on the left and a decoder block on the right. The entire framework of the Double Attention Multiscale Dense-U-Net method is displayed in Fig. 5. In order to train a deeper network without encountering vanishing gradient, DenseNets are substituted for the encoder component of the original U-Net^34,35^, as seen in Fig. 6. In order to build adaptive networks and to send lacking features to the decoder side, the recently developed skip connections are also added. The attention block of the neural network reduces the computational cost of decoding the data in each image into a vector. It also identifies which network components require more attention^18^.

The training dataset $\:A$, which consists of $\:N$ sample images, is used by the proposed model. The corresponding values are $\:A\:=$ a_1_, a_2_,., x_N_, and $\:B\:=$ b_1_, b_2_,., b_N_. Then, every ground truth pixel $\:i$ is $\:y\left[\text{0,1}\right]\:$for every sample $\:y$. In this case, a $\:224\times\:224\times\:3$ image is sent into our network, and a $\:224\times\:224\times\:1$ segmentation mask is the result. In the encoding process, an input image always passes through a combination of batch normalization, rectified linear unit (ReLU), and intense convolutional layers. The pooling layer in the transition block that follows the dense block minimizes the size of the feature map with each subsequent dense block^36–38^. To restore the feature map size to its initial value, the decoding process uses transposed convolution. Important details may be lost if the encoder path is extremely deep. To tackle this problem, UNet++^20^ presents restrictive skip connections, which groups the encoding part along with the output of up-sampling by channel concatenation.

Here, an attention technique is utilized to eliminate irrelevant data from the features, and skip connections are employed to merge several U-Net depths into a single structure. The network’s depth is s = 5, which is down-sampled five times, each time reducing the feature map’s size by half. Five down-samples later, it had the final 7 × 7 spatial feature maps. Here, the attention mechanism is used to construct a connection between the different model data at different depths. By removing unnecessary characteristics and background noise, the attention blocks make sure that only significant information advances to the following layer. The output of the encoder component is merged with the up-sampled output of the attention block using transposed convolution.

Following concatenation (dilated convolution, batch normalization, and ReLU activation), the residual block is run through the feature map, facilitating faster feature convergence. Other decoder blocks at levels $\:s\:=\:2$ to $\:s\:=\:5$ use these blocks. As shown in Eq. (17), the final segmentation map is produced by first averaging and aggregating the feature maps from all U-Net depths, then performing 1 × 1 convolution and sigmoid activation. This network was trained using the binary cross-entropy loss function grounded on the ground truth for the training images. Equation (18) displays the formula for the loss function, where $\:L$ is the loss for a prediction $\:{y}_{i}$ made with $\:N$ pixels at a specific network output:

17 $$\:b=\frac{1}{1+{e}^{-a}}$$

18 $$\:L=-\sum\:_{i=1}^{N}{b}_{i}(log{b}_{i}-\left(1-{b}_{i}\right)\text{log}\left(1-{b}_{i}\right))$$

The sigmoid function was applied with a 2D transposed convolution layer to produce the matching mask. Initialized with a random normal and a standard deviation of 0.02, this layer serves as padding. The convolution kernel is $\:5\times\:5$ with a stride of $\:2\times\:2$. The optimization technique that was selected, Adam, has a learning rate of 0.0001. Figure 5 displayed the intricate construction of the proposed model. The graphic can be made simpler by employing pointers to show the relationship between AG1 and AG4 by concatenating the relevant point from the first network with the matching point in the second network. The sigmoid function was used with a 2D transposed convolution layer to produce the matching mask. The size of the convolution kernel is $\:5\times\:5$, and its stride is $\:2\times\:2$. The optimization method Adam was selected, and it had a 0.0001 learning rate. By employing pointers to show the relationship between attention gates, the figure can be made simpler by concatenating the relevant point from the first network with the equivalent point in the second network. The attention gate is indicated by AG in this concatenated formula. The AG1 point of the second network and the AG1 of the first network, for example, are concatenated. $\:E,\:R,$ and $\:D$ stand for encoder block, residual block, and decoder block operations, respectively, that were performed on the NET1 utilizing input data $\:{X}_{in}$ in Eq. (19). This formula combines decoder path properties with an attention gate, known as AG. $\:{A}_{out1}$ is NET1’s output. In Eq. (20), the multiplication is represented as $\:{A}_{out1}$*$\:{A}_{in}$. The following is the general framework that this study suggests:

19 $$\:{A}_{out1}=\sum\:[{A}_{in}\to\:\left({E}_{1}\right)+{R}_{1}\to\:Concat({AG}_{1},{D}_{1}\left)\right]$$

20 $$\:{X}_{out2}=\sum\:[{{A}_{out}*A}_{in}\to\:\left({E}_{2}\right)+{R}_{2}\to\:Concat({AG}_{1},{AG}_{2},{D}_{2}\left)\right]$$

Ultimately, the segmented output is produced using the suggested model, which performs admirably, as demonstrated by the findings in Sect. Experiments and results.

#### Dense net

Dense connections ensure that features learned at different layers are shared across the network as in Fig. 6. This improves gradient flow during training, mitigates vanishing gradients, and enhances feature reuse, making the model more efficient in learning complex patterns. Deeper neural network when trained can upsurge a model’s accuracy, but it can also source degradation difficulties and halt the training process^39–43^. Layers $\:L$ and $\:1$ below get their respective feature maps. Unlike standard topologies, which have $\:L$ connections, an $\:L$-layer network has $\:L(L\:+\:1)/2$ direct connections. The feature maps of all preceding levels are sent to the l^th^ layer, which is represented by A_0_,.,A_(l−1)_. A_0_ = H_L_([A_0_,.,A_(l−1)_]), where $\:A0,\:A1.,\:A(l-1)$ are the feature maps concatenated in layers 0,.,l. The three procedures that make up the composite function H_l_(.) are rectified linear unit (ReLU), 33 convolution, and batch normalization (BN).

The size of feature maps is reduced in half in the pooling layers that come after the layers in normal deep CNNs. As feature maps fluctuate, the concatenation process would be erroneous. Dense encoder blocks, however, are necessary for convolutional networks and offer several advantages. For example, learning too many features can be avoided and learning time can be shortened by using fewer output dimensions in densely connected layers than in other networks. Densely connected layers guarantee extreme gradient flow, highly deep neural networks solve the vanishing gradient problem, and therefore DenseNets^21^ were employed as the encoder in our proposed technique. A first implementation of DenseNets^22^ used a 121,169,201, and 264 layer network with a k = 32 growth rate.

#### Multiscale attention in proposed double attention Dense-U-Net

**Fig. 7:**
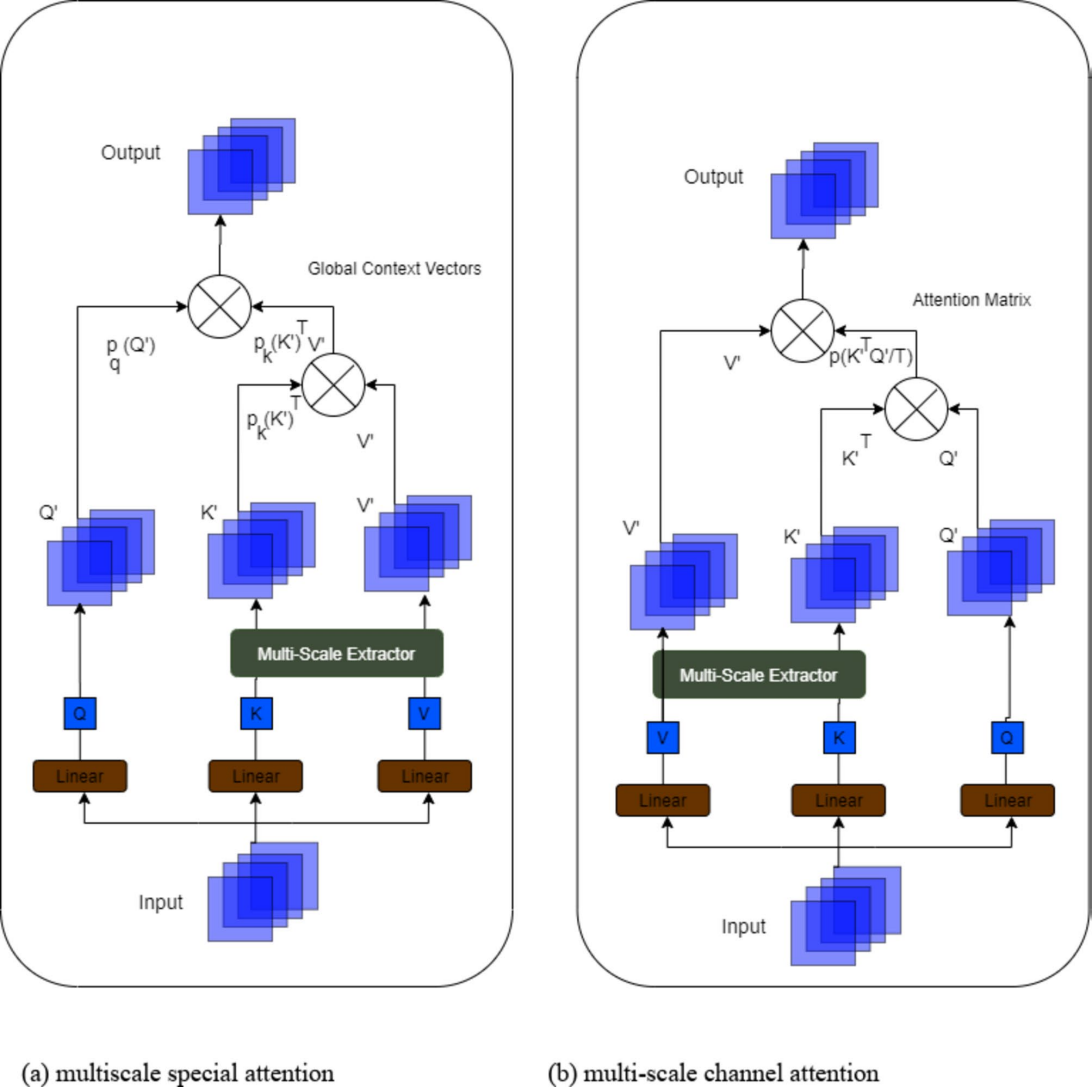
Internal details of (**a**) multiscale special attention and (**b**) multi-scale channel attention.

Multiscale attention^36^ in the Double Attention Dense-U-Net enhances the model’s ability to perform segmentation tasks by focusing on relevant features at various levels of detail and across different resolutions. This is particularly beneficial in a FL environment, where data can vary across nodes. The robustness of the model in handling multiscale features ensures better performance while maintaining data privacy. For global dependency competences, a multi-scale attention module is designed, as shown in Fig. 7(a). This can proficiently group spatial and channel features to improve feature representative capabilities using Multi-Scale Spatial Attention and Multi-Scale Channel Attention (MSCA) modules. Figure 7(b) illustrates the MSCA module, which seeks to improve semantic features according to their spatial structural similarity. Self-attention avoids secondary computational complexity by functioning along the channel dimension as opposed to the spatial dimension as each channel correlates to a semantic. In particular, channel improvement is achieved by using transposed attention^28^. Figure 7(b) illustrates our MSCA module, which seeks to improve semantic features according to their spatial structural similarity. Since every channel has a semantic equivalent, Self-attention avoids secondary computational complexity by functioning along the channel dimension as opposed to the geographical dimension. In particular, channel improvement is achieved by using transposed attention^28^:

21 $$\:C\left(Q,K,V\right)={V}_{\rho\:}\left({\widehat{K}}^{T}\widehat{Q/\tau\:}\right)$$

where *t* is a learnable temperature parameter. Here, $\:\rho(\cdot)$ denotes the application of the softmax function to each column of the computed attention matrix, and $\:\hat{K}$and $\:\hat{K}$ are $l$2-normalized *K* and *Q*, respectively. Training is stabilized by $l$2-normalization since each column of length *N* of *Q* and *K* has a unit norm. To counteract the unit norm’s diminished representational strength, *t* is also added. The attention module substitutes multi-scale $\:Q^\prime$, $\:K^\prime$, and $\:V^\prime$ for *Q*, *K*, and *V*, respectively. Rewards of multiscale attention in this model are given as follows:

- **Improved context understanding**: By including multiscale attention, the model is able to seize not only the fine details like edges and textures, but also the details like object boundaries and spatial relationships of the image.
- **Enhanced feature selection**: Multiscale channel attention aids the model to list the most important features for each image scale.
- **Robust segmentation**: The grouping of spatial and channel attention at multiple scales helps to yield more accurate results, while segmenting objects at different scales.

#### Mechanism

**Fig. 8:**
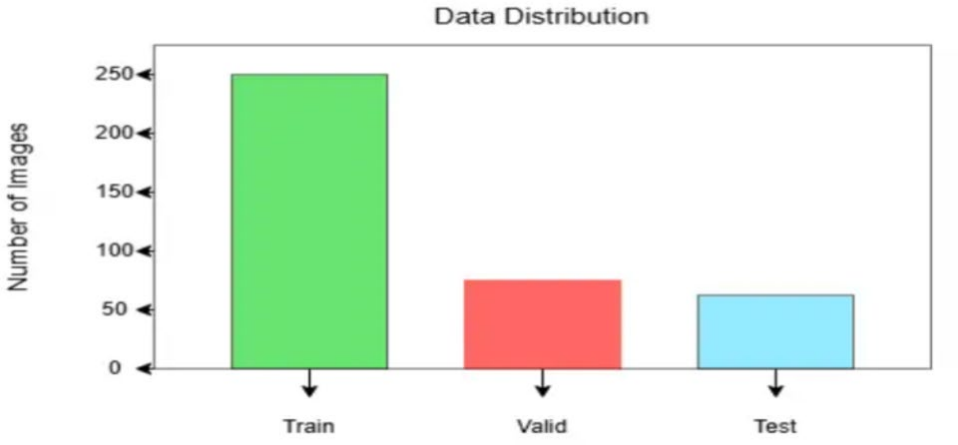
Data distribution among training, validation, and testing.

Before executing the FL system, the BraTS 2020^17^ dataset, it is divided sectionally into training, validation, and test sets. Using FL, 3 clients are demonstrated who represent the partnering institutions that will train their model on their respective parts of the dataset^37–39^. There are 249 training, 60 validation, and 45 testing data after excluding the files from the BraTS20_Training_355 folder because it has an ill-formatted name for “seg.nii” files. Figure 8 represents the data distribution.

**Fig. 9:**
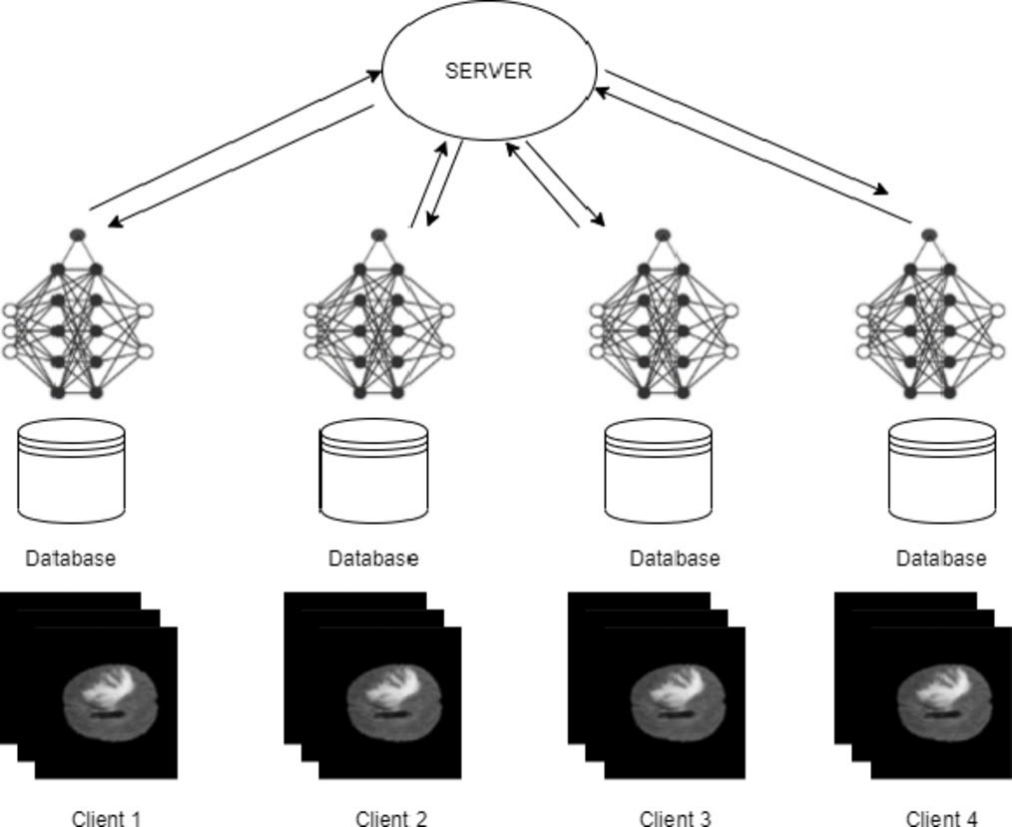
Model weights passed to the central server.

**Fig. 10:**
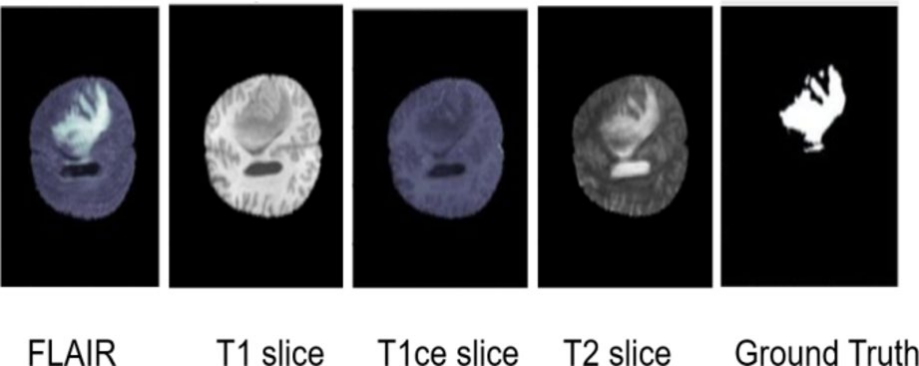
Scanned image and segmentation mask of a brain tumor.

This research involves the partition of each client’s data into $\:n$ data partitions in order to represent the quantity of clients by assigning local data to each one. This ensures that every client has access to their local training data. To train the local data for every client, U-Net architecture is implemented as mentioned above. The global model is configured first during training, and each local model receives its current weights from the main server. Before its inception, the local model is initialized, and the global model weighted values for each client are set. The weighted values of the specific model are placed into a list once the local model has been trained on the client’s dataset. Once all of the client’s model weights are known, the aggregation is conducted. In this study, the average of the weighted values is taken for the global model using the RL-FedAvg Algorithm. Each client receives the updated shared parameters from the main server for further training. After a predetermined number of federated rounds, this completes one federated round in the cycle. With this method, multi-institutional collaboration is achieved by providing each client with the parameters that they have learnt from other clients. Crucial patient data is safeguarded since the client maintains total control over their data and local training data is contained within the client’s security framework as in Fig. 9. The scanned images and the segmentation mask of a brain tumor are illustrated in Fig. 10.

## Experiments and results

### Dataset

In this research study, the BraTS 2020 Dataset is used in the experiments. Datasets such as BraTs help medical professionals evaluate, plan treatment, and track in various medical applications^40–42^. The dataset BraTS consists of T1, T1c (T1-weighted with contrast enhancement), T2, and FLAIR sequences. There are five segmentation labels like non-tumor, necrosis, edema, non-enhancing tumor and enhancing tumor. Total number of subjects are 473 and there are 5 images from each subject. Brain Tumor Segmentation Challenge (BraTS) offers a standardized dataset composed of multimodal MRI scans stored in the NIfTI format (.nii.gz). The size of each image is 224 × 224 × 150. Below is a small overview of all the different types of imaging sequences present in the BRATS 2020 dataset:

T1: This specific sequence is well-suited to depict the characteristics of native brain tissue. Longitudinal or transverse two-dimensional observations with a section’s thickness that ranges from 1 to 6 mm are ideal.

T1c: These images are also T1 weighted but with Gadolinium enhancement. They are acquired in 3D scan mode having isotropic voxel size of 1 mm.

T2: T2-weighted images show different tissue types by emphasizing variations in tissue characteristics as they are obtained through axial 2D mode having a slice thickness ranging from 2 to 6 mm.

Some of the important terms are given as follows:

- *FLAIR*: FLAIR stands for Fluid Attenuated Inversion Recovery. Compared to conventional MR imaging, FLAIR has better contrast resolutions that can reveal subtle changes in soft tissues. It encompasses three types namely axial, coronal, and sagittal two-dimensional acquisition with slice thickness ranging between 2 and 6 mm. It consists of MRI scans obtained from clinical protocol scanners at 19 institutions. Each scan has been carefully documented by experienced investigators following standard protocols. The presentations describe three main areas of interest related to brain tumors:
- *GD-enhancing tumor (ET)*: Represents an area where the tumor exhibits increased differentiation.
- *Peritumoral edema (ED)*: Refers to the area around the tumor.
- *Necrotic and non-enhancing tumor cores (NCR/NET)*: Has a central necrotic core and shallow areas giving rise to the tumor.

### Benchmarking metric

This study used various analytical parameters to investigate the model performance. Some of these metrics along with their formulas and explanations are given as below:

(i)Accuracy: Accuracy is an important measure of the effectiveness of a classification model. It shows the exact number of expected events and total events.

22 $$\:\text{Accuracy}=\frac{TP+TN}{TP+FP+TN+FN}\:\:\:\:\:\:\:\:\:$$

The Acc A, Acc B, Acc C and Acc Gobal represent the accuracies calculated for “Necrotic/Core”, “Edema”, “Enhancing” and “All classes” respectively.

- (ii) Intersection over Union (MeanIOU): A popular statistic for categorizing things up semantically problems is MeanIOU. It gauges the distance between ground truth masks and predictability.

23 $$\:\text{MeanIOU}=\frac{1}{N}{\sum\:}_{i=1}^{N}\frac{{TP}_{i}}{{TP}_{i}+{FP}_{i}+{FN}_{i}}$$

where $\:{TP}_{i}$ represents the pixel areas accurately predicted for class $\:i$ and $\:{FP}_{i}$ represents the pixel areas which is predicted as class $\:i$ but actually is from another class. $\:{FN}_{i}$ represents the pixel areas which belongs to class $\:i$ but is now predicted as some other class. Here, $\:N$ denotes the subgroup of classes. Several critical criteria were used to evaluate the UNet model’s success. MeanIOU A represents the average IOU for “Necrotic/Core”. MeanIOU B represents the average IOU for “Edema”. MeanIOU C represents the average IOU for “Enhancing”. MeanIOU Global represents the average IOU for “All classes”.

- (iii)Dice Coefficient: It is a similarity measure used to quantify the overlap between samples. It’s regularly utilized in scientific photo segmentation tasks.

24 $$\:\text{Dice}=\frac{2\times\:TP}{2\times\:TP+FP+FN}$$

- (iv)Precision: Precision quantifies how well the model predicts the future. It displays the ratio of positively predicted cases to the total number of correctly predicted cases.

25 $$\:\text{Precision}=\frac{TP}{TP+FP}$$

- (v)Sensitivity: A model’s recall, often called sensitivity, is a measurement of its ability to reliably identify every positive piece of data. This statistic aids in the computation of the population’s accurate prediction ratio relative to the entirety of data.

26 $$\:\text{Sensitivity}=\frac{TP}{TP+FN}$$

- (vi)Specificity: Specificity assesses the model’s ability to precisely identify each unwanted occurrence. It determines the proportion of all actual negative events to all accurately predicted false events.

27 $$\:\text{Specificity}=\frac{TP}{TP+FP}$$

These metrics aid in the assessment and comparison of classification as well as segmentation algorithms by offering insightful information about their performance.

(vii) GFlops: Calculate MSSA FLOPs as:

28 $$\:\text{F}\text{L}\text{O}\text{P}\text{s}=SelfAttention\:FLOPs\:(QK,\:QKV)+Downsampling/Upsampling\:FLOPs$$

Calculate MSCA FLOPs as:

29 $$\:\text{F}\text{L}\text{O}\text{P}\text{s}=Convolution\:FLOPs\:(multi-scale)+Channel\:Mixing\:FLOPs$$

To calculate GFLOPs use the following steps:

1. Using the formulas given in (28) and (29) calculate the FLOPs per layer/block.
2. Calculate the sum of FLOPs throughout all layers given in the model for a given input.
3. It is then converted into to GFLOPs by dividing by 10^9^.

### Validation and model training

Participants submit model outputs for the test set, and the BraTS evaluation server calculates metrics based on hidden ground truth. The proposed model is robust to variations in intensity, resolution, and noise. This model ensures that both tumor and non-tumor regions are well classified, avoiding over-segmentation or under-segmentation.

Diverse patient demographics and imaging protocols ensure model robustness. A conventional method that entailed dividing the data into sets for both training and validation was used to train and validate the FL model. While the training set was used to modify the model’s parameters, the validation set evaluated the model’s performance. Each user used their information to train its model, while the server used a weighted average technique to aggregate the model adjustments. The model’s performance was assessed using the meanIOU, accuracy, precision, sensitivity, specificity, and dice coefficient. Each local model was trained using validation data, which is also used to assess each model’s efficiency. Using the testing dataset, the final test is conducted following completion of all training.

### Hyperparameter tuning

In this work, thorough testing and hyperparameter fine-tuning were critical for optimizing the performance of deep learning models. By carefully experimenting with different batch sizes, learning rates, and optimizer configurations, the most effective combination is found that produced better outcomes^30^. The batch size of 100 samples, a learning rate of 1e-3, and the Adam optimizer^31,32^ were chosen for their ability to minimize training loss while increasing model accuracy. The Table 2 describes the hyperparameters. Furthermore, using callback methods like ReduceLROnPlateau improved the model’s training adaptability. By dynamically modifying the learning rate in response to validation loss patterns, the model may fine-tune its parameters more effectively, accelerating convergence and optimizing overall performance.

**Table 2:** Hyperparameters for brain tumor segmentation.

Hyperparameter	Value
Model Architecture	UNet
Activation Function	ReLU
Kernel Initializer	He Normal
Dropout Rate	0.2
Optimizer	Adam
Learning Rate	0.001
Loss Function	Categorical Crossentropy
Callbacks	ReduceLROnPlateau

### Model performance

In this section, initially the individual federated model techniques are compared with each other. Later on, the model is compared with the centralized model using accuracy (Acc) and MeanIOU as performance metrics. In the Table 3, the different FL techniques are compared. FedProx (Federated Proximal), FedProx-even (Federated Proximal with Even Data Distribution), FedNova (Federated Averaging with Novelty Detection), FedWeightedAvg (Federated Weighted Averaging), FedBN (Federated Batch Normalization)^16^, are compared with our proposed model which uses RL-FedAvg (Federated Averaging). The proposed methodology was able to produce the best results.

**Fig. 11:**
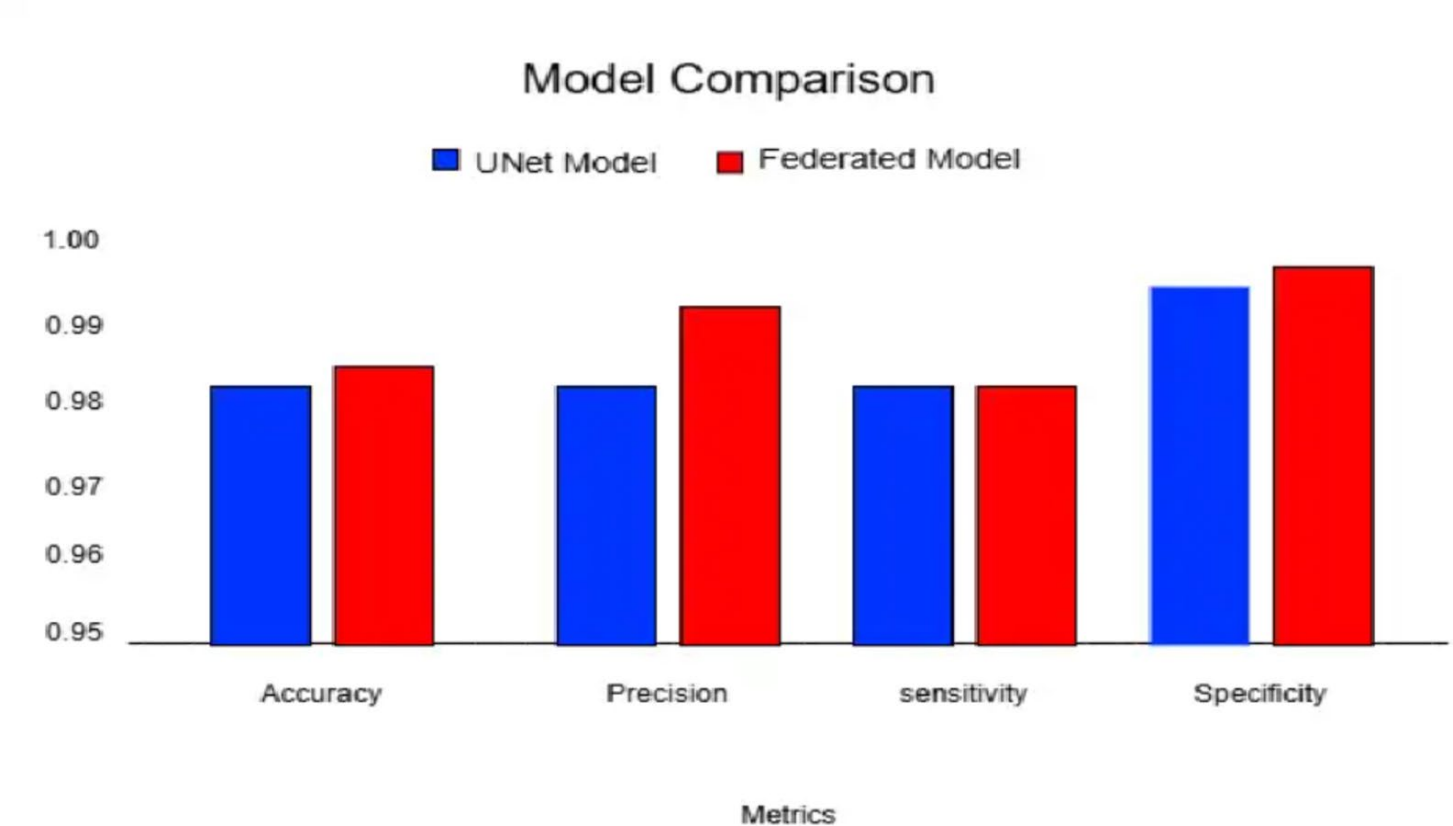
Performance scores of the conventional U-Net and the federated model.

As in Table 4, the proposed UNet Model, exhibits impressive performance across the board. It correctly classifies most of the pixels in the classification test with an accuracy of 98.24%. The MeanIOU score of 83.23% highlights its expertise in defining object boundaries and overlapping areas, which is important for accurate classification. Furthermore, the proposed UNet model has 98.24% accuracy. Its sensitivity skillfully identifies positive cases, and reduces false positives and false negatives. On the other hand, the FL model exhibits an accuracy at 98.56%, and a precision of 99.27%. However, its MeanIOU score is slightly lower at 82.27%. Even so, its 98.31% sensitivity means it can capture the best cases accurately. Furthermore, with a specificity of 99.74%, the federated model excels in correctly detecting bad conditions. Table 4 describes the central vs. FL model performance. Figure 11 shows the performance scores of the model.

In summary, both models exhibit similar performance, and each has its strengths. The federated model is slightly ahead in accuracy and precision and also provides privacy of data to the clients. This proves FL is a method to be used in real-life scenarios. Both models excel in specificity, highlighting their effectiveness in accurate classification tasks. Both models can produce accuracy above 98% and show great results.

In the proposed Mixed-FedUNet model, the global model is constructed using the Federated Averaging algorithm, a commonly used approach in federated learning. This algorithm involves combining the weights of local models trained on datasets and aggregating them to create a model operating on a global scope. At the main server, the received weights from all participating institutions are aggregated using the RL-FedAvg algorithm. This aggregation process typically involves computing the average of the weights across all local models, weighted by the number of samples or another parameter representing the contribution of each local model. The resulting aggregated weights represent the global model.

**Table 3:** Performance analysis of different FL techniques.

Model	Acc A	MeanIOU A	Acc B	MeanIOU B	Acc C	MeanIOU C	Acc Global	MeanIOU Global
FedProx	98.6	71.8	99.2	72.5	97.9	79.7	98.3	75.7
FedProx-even	98.7	73.2	98.4	80.1	98.0	72.9	98.4	73.4
FedNova	99.5	72.0	98.2	81.5	97.8	70.3	98.2	79.2
FedWeightedAvg	98.8	80.3	98.6	78.9	98.2	79.7	97.5	80.6
FedBN	98.9	76.2	99.6	80.2	99.6	79.7	98.5	79.7
**Mixed-FedUNet**	98.7	82.2	99.6	83.2	98.2	83.2	98.5	82.2

**Table 4:** Central vs. FL model performance.

Model	Accuracy	MeanIOU	Precision	Sensitivity	Specificity
UNet Model	0.9824	0.8273	0.9824	0.9824	0.9941
Federated Model	0.9856	0.8227	0.9927	0.9831	0.9974

**Fig. 12:**
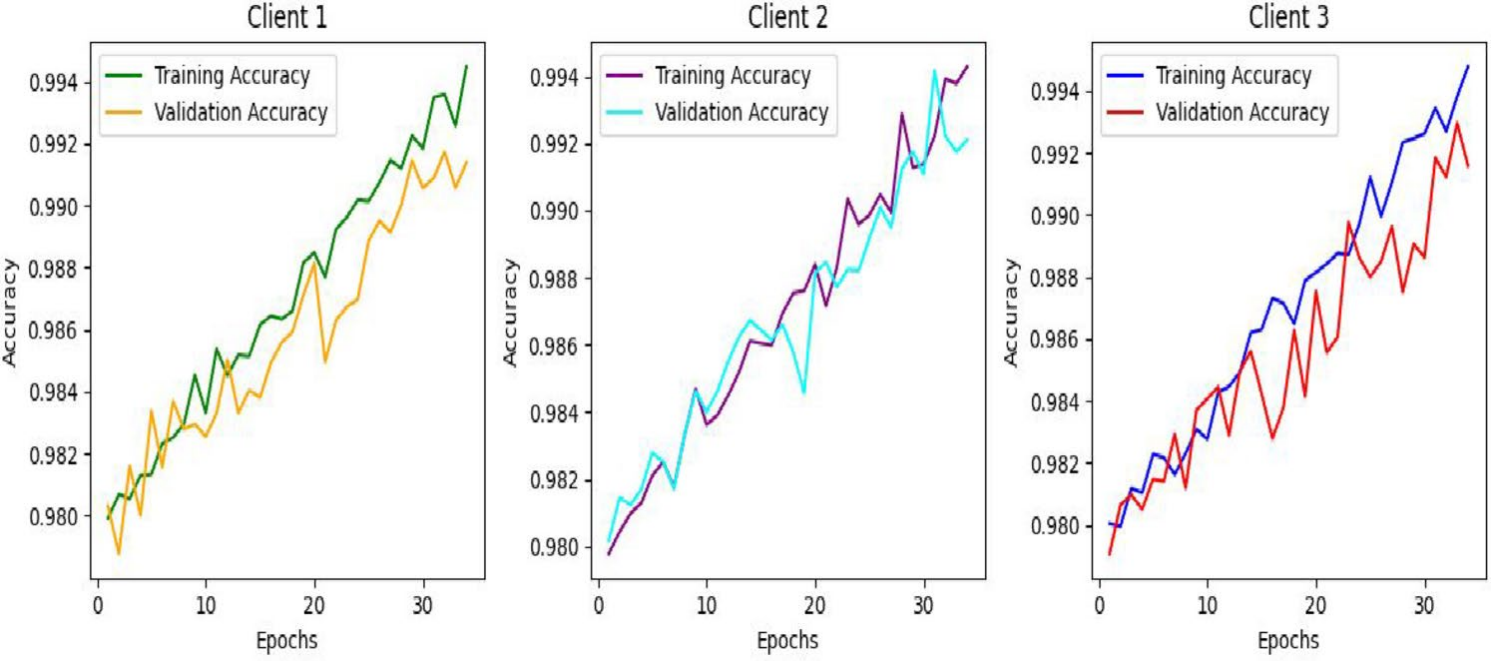
Training and validation accuracy of clients in the 1st epoch.

The above graph shows increasing accuracy as the model trains on the particular dataset with the number of epochs and time. In the training and validation, each client can achieve an accuracy of almost 99%. Figure 12 shows the training and validation accuracy of the clients.

### Impact of federated learning on data privacy

FL tackles security and privacy concerns related to data in medical imaging applications. The impact of FL on the security and confidentiality of data is assessed in this section, along with any potential leaks and security issues during data communication with the client and central server. A model inversion attack is one of the main types of attacks that might occur in a FL ecosystem. An example of a privacy attack is a model inversion attack, which looks at the parameters or outputs of a machine learning model in an effort to deduce sensitive information about specific data points. To prevent model inversion attacks, other techniques such as differential privacy are used, which adds noise to model updates in aggregation and deprives attackers of specific information about individual contributions. RL-FedAvg algorithm effectively achieves good prevention against model inversion attacks.

### Classification report

**Fig. 13:**
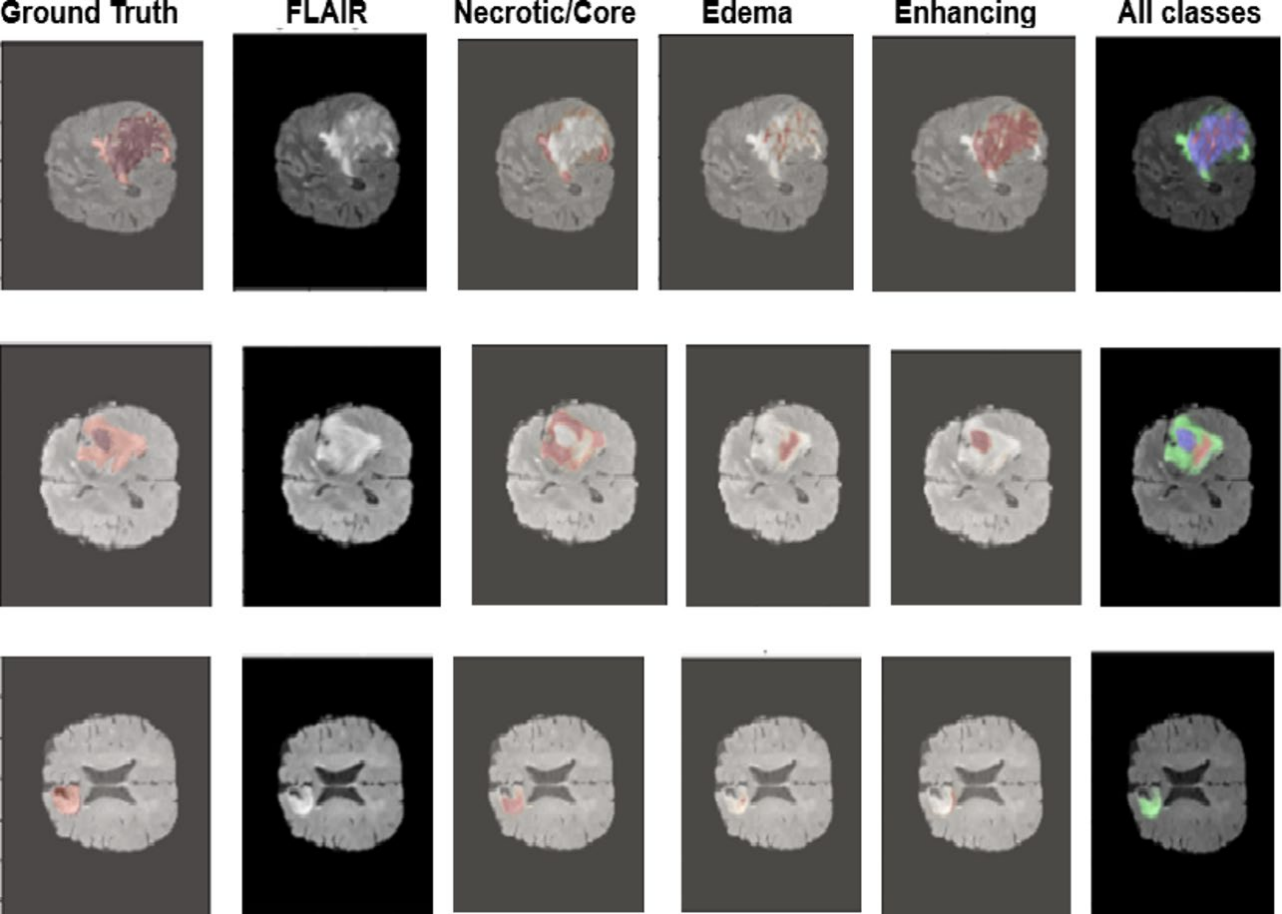
Predicted segmentation using Mixed-FedUNet.

Figure 13 represents the predicted segmentations. The GD-enhancing tumor is annotated as ET (label 4), the peritumoral edema as ED (label 2), and the necrotic and non-enhancing tumor core as NCR/NET (label 1). The segmentation results span several classes. Some predictions target specific tumor components, while the “All classes” image displays a comprehensive segmentation that encompasses structures and healthy tissue. The “NECROTIC/CORE predicted” image highlights the necrotic core, which is crucial for understanding the variation in tumors and evaluating treatment response. The “EDEMA predicted” picture, which is essential for evaluating tumor invasion and surrounding tissue damage, defines peritoneal edema. Ultimately, the “ENHANCING predicted” image identifies enhancing regions suggestive of active tumor growth, assisting in the guidance of treatment methods such as targeted therapy and surgical removal. Table 5 describes the classification report indicating the good performance of the proposed model.

**Table 5:** Classification report.

Class	Precision	Recall	F1-score
All classes	1.00	1.00	1.00
Necrotic/core	0.93	0.93	0.89
Edema	0.97	0.95	0.86
Enhancing	0.93	0.98	0.86

### Ablation studies

Ablation studies are conducted to assess the requirement and efficacy of the suggested enhanced operation.

#### Effect of different scales

First, the impact of the proposed multi-scale information interaction on the performance of the model is tested. As one can see from below Table 6, the results are compared. The number 1 × 1 denotes no multi-scale information sharing. The table clearly shows that segmentation performance is improved through inclusion of information from a few scales. This reveals that multi-scale information helps in achieving effective interaction which is not possible in single scale. Furthermore, when the data of all four scales interact with each other, this model’s global analysis is more apt, and it produces the highest Dice score of 93.28%. First, it assesses how the suggested multi-scale information interaction will affect the performance of the model. Table 6 shows the comparison of the results. A value of 1 × 1 represents no multi-scale information sharing.

#### Effect of double attention multiscale Dense-U-Net

The segmentation performance of the proposed Dual Multi-Scale Attention (DMSA)^43,44^ module is evaluated as in Table 7. From the table, it can be seen that our optimized UNet reduces the calculations and parameters with very low performance loss. Increased focus on needed areas was achieved by introducing the Double Attention Multiscale DenseNet module within the efficient UNet. The model still demonstrated low model complexity while surpassing vanilla UNet performance by 1.83%. Model-wide analysis indicated better performance by utilizing data from all four scales, as indicated by the Dice score of 93.28%.

#### Effect of last Convolution block

Because the convolutional kernel is fixed, convolutional processes can easily introduce redundant noises during deep semantic extraction. The problems mentioned above can be well-avoided with the help of a global attention mechanism, which adaptively chooses features for aggregation based on their importance. Double Attention Multiscale DenseNet-UNet thus considers removing the deepest convolutional block in UNet in the ablation study. The results of segmentation are represented in Table 8. From the table, it is evident that once the last convolutional block is removed, model complexity decreases, and the Dice score increases by 0.22%.

#### Effect of MSSA and MSCA

Table 9 is the disassembly of a Double Attention Multiscale Dense-U-Net, where the relevance of MSSA and MSCA are evaluated. For fairness, the MSSA and MSCA, when compared independently, are doubled in number. The MSSA and MSCA had collaborated more effectively as compared when they worked individually, as shown in the table. This implies the global information could be used even more intensively in both spatial and channel dimensions to boost performance. Further, it is obvious that the channel attention MSCA outperforms its spatial attention MSSA counterpart in competitiveness sense, mainly because MSCA exploits traditional similarity attention mechanism.

**Table 6:** Effect of MSSA and MSCA.

Size of kernel	#Params (M)	#GFLOPs	Dice (%)
1 × 1	21.52	31.81	92.32
3 × 3	21.92	32.58	93.02
5 × 5	21.95	32.65	92.83
7 × 7	21.99	32.75	92.56
All scales	21.89	32.54	93.28

**Table 7:** Performance of the Multi-Scale attention.

Techniques	#Params (M)	#GFLOPs	Dice (%)
Vanilla UNet	32.04	37.98	91.45
Proposed UNet	9.48	15.44	91.12
**Proposed UNet with Double Attention Multiscale DenseNet**	21.89	32.54	93.28

**Table 8:** Performance of the proposed work with/without the last convolutional block.

Techniques	#Params (M)	#GFLOPs	Dice (%)
With last Conv. block	28.72	33.88	93.06
Without last Conv. block	21.89	32.54	93.28

**Table 9:** Performance of MSSA and MSCA.

Methods	#Params (M)	#GFLOPs	Dice (%)
MSSA²	21.89	32.54	92.75
MSCA²	21.89	32.54	93.05
MSSA + MSCA	21.89	32.54	93.28

### State-of-the-art comparison

The Table 10 is a curated selection of recent research initiatives in FL, particularly in healthcare applications. Each entry in the table reflects the breakthroughs that have occurred in FL techniques. For example, Lee et al.^20^ presented the FML (Federated + ML) model and showed its efficacy in predicting fetal health with an astonishing 99.06% accuracy. This demonstrates the possibility of combining federated learning with classic machine learning techniques to improve predictive capacities in healthcare. In this work, the proposed methodology uses a federated averaging algorithm to enable model aggregation and updates across three clients. The Mixed-FedUNet model secures patient data while achieving high segmentation accuracy by training the model individually on each client’s dataset and only exchanging updates to the model with the main server. With an accuracy of 98.56%, the model demonstrates its ability to effectively identify brain tumor boundaries using medical imaging data.

**Table 10:** State-of-the-art comparison with other focus areas.

Ref.	Year	FL Model Used	Focus Area	Best Accuracy (%)
^22^	2023	FeAvg-CNN, MobileNet	Breast Cancer Classification	97.91
^23^	2023	Ensemble Federated Learning (FL)	Lung Cancer Diagnostics	89.63
^25^	2023	PrivateFL	Privacy-Preserving FL	93.30
^26^	2023	Collaborative FL	COVID-19 Screening from Chest X-rays	97.00
^28^	2022	Differentially private FL	Histopathology Image Analysis	85.00
^30^	2024	FedCure	Intelligent Healthcare Applications	90.02
**Proposed**	**2024**	**Mixed-FedUNet**	**Brain tumor segmentation**	**98.56**

**Table 11:** Performance comparison with the other models for brain image segmentation.

Reference	Year	FL Model Used	Best Accuracy (%)	DSC
^45^	2024	Federated U-Net	83.11	80.23%
^46^	2020	Centralized U-Net	80.23	78.32%
^47^	2023	Personalized Federated U-Net	87.14	85.25%
^48^	2024	Attention Federated U-Net	88.54	85.45%
^49^	2021	Multi-scale Federated U-Net	89.34	85.42%
**Proposed**	**2024**	**Mixed-FedUNet**	**98.56**	**93.28%**

The Double Attention mechanism in Mixed-FedUNet allows for focused attention on relevant features at multiple stages of the model, refining feature extraction and segmentation. This dual-attention structure better captures complex tumor characteristics compared to single attention mechanisms in models like Attention Federated U-Net. The dense connections in Mixed-FedUNet facilitates efficient feature reuse and improves gradient flow, reducing the vanishing gradient problem often encountered in deep models. This results in better model convergence and stability in federated training. Dense architecture enhances feature reuse and model efficiency. Mixed-FedUNet offers consistent segmentation quality across clients and the federated setup in Mixed-FedUNet protects privacy while ensuring accuracy as in Table 11.

**Fig. 14:**
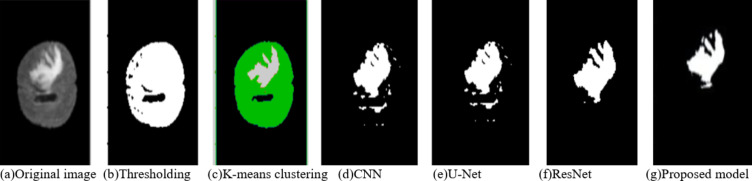
Comparison of the segmentation outputs from the proposed model compared with baseline models.

Figure 14 represents the comparison of the proposed model with the other existing methods for segmentation. The proposed model achieves improved segmentation when compared to the clustering, thresholding, KNN, CNN, ResNet and Unet. In general, Double Attention-based Multiscale Dense-U-Net normally surpasses the traditional methodologies of thresholding, k-means clustering, and even that of CNN-based techniques in both terms of accuracy and robustness when applied for brain image segmentation. It incorporates a multi-scale architecture coupled with double attention mechanisms to effectively seize the spatial as well as channel-wise dependencies, giving an improved extraction of the feature and better segmentation capability for complex structures of a brain. Thresholding and k-means clustering techniques generally perform poorly in picking fine-grained details because of simpler assumptions of these methods and failure to cope with the variability in brain-image complexities. CNNs can also facilitate segmentation by learning hierarchical features but may not excel, especially in fine details due to noisy or very volatile data. While improving the segmentation, U-Net’s encoder-decoder architecture does not have high resolution on important features compared to the attention mechanisms of Double Attention-based Multiscale Dense-U-Net. Therefore, deep residual connections in ResNet would provide excellent segmentation while potentially requiring more computational resources and fine-tuning. The targeted attention layers from Double Attention-based Multiscale Dense-U-Net achieve better performance with fewer parameters. Overall, Double Attention-based Multiscale Dense-U-Net offers a more holistic, accurate, and computationally efficient approach in the case of complex and noisy brain imaging data.

The accuracy obtained during segmentation is measured using Dice Coefficient. Without privacy mechanisms also the segmentation accuracy is good, but it may vary for different datasets. With differential privacy-based technique there is improved consistency in accuracy. RL-fedAvg optimizes the learning process without compromising the privacy. The model inversion attack resistance is measured using PSNR. When there is model inversion attack, the images can be reconstructed when there is no privacy. Privacy risks are lesser resulting in low PSNR ratio for the proposed RL-FedAvg. The privacy protection is measured by means of L2 norm of gradients. The L2 norm of gradients is significantly lesser in case of differential privacy and RL-FedAvg which helps in protecting the data from reconstruction. In RL-FedAvg the convergence rate is 55 epochs which optimizes the overall training process as in Table 12.

**Table 12:** Performance analysis in terms of privacy.

Methods/Metric	No privacy	Differential privacy	RL-FedAvg
Dice Coefficient	0.83	0.86	0.93
PSNR	35.3dB	12.6dB	10.4dB
L2 Norm of Gradients	1.3	0.7	0.6
Convergence Rate	60 epochs	70 epochs	55 epochs

Cross evaluations are performed on the 150 samples chosen from other datasets, such as the BraTS 2021 (https://www.kaggle.com/datasets/dschettler8845/brats-2021-task1) and BraTS 2023 (https://www.kaggle.com/datasets/shakilrana/brats-2023-adult-glioma) datasets^50^, in order to more accurately evaluate the flexibility and generalization capabilities of the model in various clinical settings. Table 13 provides a summary of the quantitative results from external validation. The robust generalization on new data is demonstrated by the cross-dataset validation results, which show comparable performance. This demonstrates that even with entirely new data, the model is effective at segmentation.

**Table 13:** Evaluation results obtained from brats 2021 and brats 2023 datasets.

Dataset	#GFLOPs	Dice Coefficient	Accuracy (%)	#Params (M)
BraTS 2021	33.44	94.5	98.23	22.45
BraTS 2023	34.32	94.6	98.33	23.67

The proposed approach has good data privacy whereas the other state-of-the art techniques like UNet, UNet + + and attention-UNet using centralized data processing techniques have limited privacy. The proposed approach can handle heterogenous data efficiently than the other centralized approaches used in the state-of-the-art techniques. The proposed approach effectively captures the global and local features using the Double Attention-based Multiscale Dense-U-Net compared to the other state-of-the art algorithms. The Multiscale Dense-U-Net can effectively handle multiple tumor shapes and sizes compared to the conventional UNet, UNet + + and attention-UNet algorithms. The proposed approach is ideal for privacy sensitive applications compared to the other state-of-the-art algorithms.

The RL-FedAvg model integrates reinforcement learning with federated learning for optimized aggregation over different and decentralized datasets further improving privacy and generalization in the task of brain tumor segmentation. The Double Attention-based Multiscale Dense-U-Net improves the accuracy of segmentation by utilizing global and local information via dense connection and double attention mechanism. Collectively, these models tackle crucial problems of the current paradigms including, non-IID data distribution problems. This proposed model improves the accuracy, scalability and privacy of medical image analysis methods. Still some risks exist like gradient leakage, model inversion, and poisoning attacks. Privacy can be further improved by techniques like differential privacy, secure aggregation, and robust federated learning methods in future works.

The computational complexity of the proposed model is given as:

30 $$\:O(N\times\:E\times\:D\times\:F\times\:H\times\:W\times\:{K}^{2})+O(T\times\:N\times\:P)+O({T}_{RL}\times\:N\times\:P)$$

Here:

- N indicates the total number of clients.
- E represents the number of epochs.
- D indicates the size of dataset.
- H×W represents the image resolution.
- F represents the total number of feature maps produced.
- K indicates the size of the kernel.
- P is the total number of parameters.
- T represents the total number of rounds in federated learning.
- $\:{T}_{RL}$ represents the number of iterations

To further reduce the computational complexity, model compression and improved aggregation approaches can be incorporated in future work. To further improve the scalability, techniques like gradient compression, dynamic client selection, and communication constrained federated learning can be applied on the proposed model. The proposed approach is ideal for privacy sensitive applications compared to the other state-of-the-art algorithms.

Modifying the RL-FedAvg model for applications like multi-organ segmentation or multi-modality imaging would take the collaborative health-care to the next level in terms of privacy preserving AI. The multi-organ segmentation or multi-modality imaging AI, known as the Double Attention based Multiscale Dense-U-Net, is also applicable for histopathology and multi-class segmentation, which allows for accurate relevancy across different types of images. Applying these models to combine large-scale, decentralized medical imaging could result in federated attention networks. Reinforcement learning strategies, including hierarchical RL or dynamic optimization of reward prediction, will obviously help improve the model’s performance and accuracy. These are technologies that can be a game-changer for diagnosis, treatment and medical image analysis in general.

Future research on the Double Attention-based Multiscale Dense-U-Net for brain image segmentation could optimize attention mechanisms for more precise pathological feature extraction. Also, multi-modal imaging can be used for all-inclusive analysis, which enhances real-time processing to make it clinically deployable. Semi-supervised learning techniques and improved model interpretability could also reduce dependence on large amounts of labeled data and increase trust in its clinical application. Finally, cross-dataset testing and integration with decision support systems could further validate its relevance and adaptability in the clinical setting. Some of the possible enhancements for Double Attention-based Multiscale Dense-U-Net in brain image segmentation could be fine-tuning the model for other medical imaging tasks, like organ segmentation or lesion detection. Reinforcement learning strategies within a federated learning framework could enable collaborative learning from distributed healthcare data while maintaining patient privacy. Moreover, combining adaptive reinforcement mechanisms could improve the accuracy of segmentation over time, thereby enhancing real-world clinical performance of the model.

The brain image segmentation by using Double Attention-based Multiscale Dense-U-Net with reinforcement-based federated learning improves brain tumor segmentation. It is about making the analysis more efficient, accurate, and privacy-preserving. It allows the diagnosis to be enhanced and supports personalized treatment planning while being able to work collaboratively between health institutions without breaching patient data confidentiality. This can support integration into clinical workflows to aid radiologists with real-time, reliable segmentations to enhance AI-driven medical decision-making.

## Conclusion

The use of FL allows for model training across several datasets without requiring data sharing, ensuring secrecy. FL models outperform previously unreported data by leveraging heterogeneous data from many places, pointing to promising advances in brain tumor detection and treatment planning. Furthermore, when a larger dataset is available for training, the model receives a wider variety of features and parameters, thus increasing the ability to generalize and make more accurate predictions. Despite FL’s intrinsic privacy protections, it remains prone to possible dangers, particularly information leakage via model upgrades. Federated Averaging (FedAvg) combined with Reinforcement Learning (RL)-enabled Double Attention-based Multiscale Dense-U-Net introduces several key advantages for enhanced brain tumor image segmentation. This approach influences the strengths of FL for privacy preservation, reinforcement learning for dynamic optimization, and advanced deep learning architectures for accurate image segmentation. Client-side data augmentation and block chain can be used to improve model robustness in future works. Each client can generate synthetic brain tumor images by applying transformations such as rotation, zooming, and intensity adjustments. This would expand the diversity of the local datasets without violating privacy regulations. For rare tumor types, generative techniques such as GANs (Generative Adversarial Networks) could also be used to generate synthetic brain MRI images, enriching the training data.

## Data Availability

The BraTS 2020 dataset can be obtained from https://www.kaggle.com/datasets/awsaf49/brats20-dataset-training-validation. The BraTS 2021 dataset can be obtained from https://www.kaggle.com/datasets/dschettler8845/brats-2021-task1. The BraTS 2023 dataset can be obtained from https://www.kaggle.com/datasets/shakilrana/brats-2023-adult-glioma.
